# Whole-Exome Sequencing Reveals Novel Candidate Driver Mutations and Potential Druggable Mutations in Patients with High-Risk Neuroblastoma

**DOI:** 10.3390/jpm14090950

**Published:** 2024-09-08

**Authors:** Natakorn Nokchan, Praewa Suthapot, Pongsakorn Choochuen, Natthapon Khongcharoen, Suradej Hongeng, Usanarat Anurathapan, Komwit Surachat, Surasak Sangkhathat, Thai Pediatric Cancer Atlas (TPCA) Consortium

**Affiliations:** 1 Department of Biomedical Sciences and Biomedical Engineering, Faculty of Medicine, Prince of Songkla University, Songkhla 90110, Thailand; natakorn.n@psu.ac.th (N.N.); praewa.sut@mahidol.ac.th (P.S.); pongsakorn.c@psu.ac.th (P.C.); 6510330010@psu.ac.th (N.K.); komwit.s@psu.ac.th (K.S.); 2 Translational Medicine Research Center, Faculty of Medicine, Prince of Songkla University, Songkhla 90110, Thailand; 3 Division of Hematology and Oncology, Department of Pediatrics, Faculty of Medicine Ramathibodi Hospital, Mahidol University, Bangkok 10400, Thailand; suradej.hon@mahidol.ac.th (S.H.); usanarat.anu@mahidol.ac.th (U.A.); 4 Center of Multidisciplinary Technology for Advanced Medicine (CMUTEAM), Faculty of Medicine, Chiang Mai University, Chiang Mai 50200, Thailand; 5 Department of Surgery, Faculty of Medicine, Prince of Songkla University, Songkhla 90110, Thailand

**Keywords:** neuroblastoma, tumor-only, whole-exome sequencing, somatic variants, mutational signatures, tumor mutational burden, cancer driver gene, druggable mutations

## Abstract

Neuroblastoma is the most prevalent solid tumor in early childhood, with a 5-year overall survival rate of 40–60% in high-risk cases. Therefore, the identification of novel biomarkers for the diagnosis, prognosis, and therapy of neuroblastoma is crucial for improving the clinical outcomes of these patients. In this study, we conducted the whole-exome sequencing of 48 freshly frozen tumor samples obtained from the Biobank. Somatic variants were identified and selected using a bioinformatics analysis pipeline. The mutational signatures were determined using the Mutalisk online tool. Cancer driver genes and druggable mutations were predicted using the Cancer Genome Interpreter. The most common mutational signature was single base substitution 5. *MUC4*, *MUC16*, and *FLG* were identified as the most frequently mutated genes. Using the Cancer Genome Interpreter, we identified five recurrent cancer driver mutations spanning *MUC16*, *MUC4*, *ALK*, and *CTNND1*, with the latter being novel and containing a missense mutation, R439C. We also identified 11 putative actionable mutations including *NF1* Q1798*, Q2616*, and S636X, *ALK* F1174L and R1275Q, *SETD2* P10L and Q1829E, *BRCA1* R612S, *NOTCH1* D1670V, *ATR* S1372L, and *FGFR1* N577K. Our findings provide a comprehensive overview of the novel information relevant to the underlying molecular pathogenesis and therapeutic targets of neuroblastoma.

## 1. Introduction

Neuroblastoma (NB) is the most common solid tumor in infants with a median age of 18 months; however, it can occur at any time during childhood [1]. It is a common malignancy of the sympathoadrenal lineage of the neural crest. The tumor can originate anywhere along the sympathetic chain but is most frequently found in the adrenal medulla and paraspinal ganglia. Metastatic disease has been observed in the distant lymph nodes, bones, bone marrow, liver, and skin [2]. Global epidemiological studies of pediatric cancers in the United States [3,4,5] and Europe [6] over the last two decades have identified the prevalence of neuroblastoma in patients at an annual rate of 6–7.5%. In Thailand, the reported incidence of neuroblastoma between 1990 and 2011 was 3–5 per million people/year. Calculations based on reported trends in Thai pediatric cancers indicated that NB accounted for 5% of all pediatric cancers or 14.7% within the group of solid tumors [7,8]. Although the survival rate of solid tumors over the study period showed significant improvement compared with the second and first decades, the survival for central nervous system tumors, NB, and bone tumors did not change [9]. The 5-year-related survival rate of patients with NB ranged from 20% to 70%. The low survival rate observed in neuroblastoma is attributed to the presence of the disease at an advanced stage, multivariate prognostic factors, and limited options for curative treatments [9,10,11]. Reportedly, the 5-year overall survival (OS) of patients with high-risk disease was less than 50%, emphasizing the need for novel immunotherapeutic approaches [12]. Chemoresistance at the beginning or during the treatment influences the prognosis of high-risk patients and has become a major problem in NB treatment [13]. The identification of chemoresistance biomarkers and alternative treatments to overcome chemoresistance would be beneficial.

The disease is noteworthy for its broad spectrum of clinical behavior and can be classified into three risk groups (low, intermediate, and high risk) based on age, MYCN status, and histology, as defined by the International Neuroblastoma Staging System (INSS) [2,14]. The more advanced tumor-staging system provided by the International Neuroblastoma Risk Group (INRG) implements an image-based staging system and classifies patients according to their pattern of localized or metastatic disease [15]. Recently, the revised Children’s Oncology Group (COG) risk classification system has been developed to accurately classify risks and stratify treatments for eligible patients. This includes incorporating the INRG staging system and segmental chromosome aberrations, which serve as a novel prognostic biomarker [16]. The signs and symptoms of NB are highly variable and depend on age, primary tumor site, presence of metastasis, and, occasionally, associated paraneoplastic syndromes. Risk-stratified therapy, as opposed to intensive multimodal therapy for high-risk NBs, has facilitated a reduction in therapy in children with low- and intermediate-risk diseases [17]. For patients with high-risk NB, although high-intensity multimodal treatment comprising surgery, chemotherapy, radiotherapy, high-dose chemotherapy with autologous hematopoietic stem cells, and immunotherapy is administered, poor prognosis and long-term sequelae are still observed [18]. While the risk-group classification system can provide a platform for uniformly defining risk, recent approaches targeting genetic pathways and the tumor microenvironment hold promise for future curative treatments.

The discovery of robust genomic and molecular biomarkers has provided a framework for more precise prognostication and refined therapeutic approaches aimed at a better long-term quality of life [19,20]. Tumor-specific genetic markers and histopathological assessments are crucial determinants of treatment. Many genetic features of NB, such as amplification of the MYCN proto-oncogene (20–30% of patients), chromosomal aberrations including 1p loss (~30%), 11q loss (~40%), and 17q gain (~50%), and tumor cell ploidy, have been identified, revealing their potential as powerful predictors of response to therapy and outcomes [2]. However, these genetic factors cannot be used to elucidate the mechanisms underlying the malignant tumor’s transformation and progression. Thus, efforts have focused on using molecular biomarkers to fine-tune the relationship between genetic abnormalities and novel therapeutic options [21,22]. According to pan-cancer genomic analysis, unlike adult cancers, most of the commonly mutated genes in pediatric cancers are mutually exclusive across tumor types.

The major defective pathways include the downstream components of the MAPK, cell cycle, and DNA repair pathways [23]. Heritable mutations in *ALK* and *PHOX2B* have been identified as disease-causing mutations in familial NB [24]. Genetic alterations can be found in both familial and sporadic NBs, presenting a promising therapeutic target for *ALK*. Several comprehensive analyses of genomic aberrations in NB have been conducted using various approaches. Somatic mutations frequently detected in NB include *ALK*, *PTPN11*, *ATRX*, *MYCN*, *NRAS*, *TERT*, and *ARID1A*/*ARID1B*, with potential associations with age, progression, and prognostic factors [24,25,26,27,28,29]. Mutations in *TP53*, a highly recurrent mutated gene in over 50% of all human cancers, are rarely found in NB tumors [30]. Thus, the challenge over the next decade is to determine how to translate this information into more effective and less toxic therapies for these patients.

Despite major improvements in next-generation sequencing, the genetic study paradigm for high-risk NB tumors still shows little improvement in clinical outcomes. In this study, we performed whole-exome sequencing of tumors from 48 patients with NB. Somatic alterations, tumor mutational burden, mutational signature, the protein–protein interaction (PPI) network, enriched Gene Ontology (GO) terms, and Kyoto Encyclopedia of Genes and Genomes (KEGG) pathways corresponding to somatic mutations were analyzed. Additionally, we attempted to identify alterations in cancer drivers as targets to counteract chemoresistance and predict drug responses based on these candidate cancer drivers. The comprehensive characterization of NB tumors should shed light on the molecular pathogenesis of NB and provide potential targets for predicting drug responsiveness in targeted therapy for patients. These drug targets offer a novel therapeutic strategy for chemoresistant patients.

## 2. Materials and Methods

### 2.1. Patients and Samples

Forty-eight fresh-frozen tumor samples were retrospectively collected from the Biobank of the Translational Medicine Research Center, Faculty of Medicine, Prince of Songkla University, and the Ramathibodi Tumor Biobank, Faculty of Medicine, Ramathibodi Hospital, Mahidol University. Surgical specimens were obtained from patients with NB admitted to the Songklanagarind Hospital between 2007 and 2015 and Ramathibodi Hospital between 2019 and 2021, all of whom were under 15 years of age. These patients were initially diagnosed with NB based on histologically confirmed tumor samples examined by pathologists. The samples were then dissected and stored at −80 °C in the Biobank. Clinical data were retrieved from the hospital information systems. The INSS and INRG scores for each patient were obtained from medical records. The pre-treatment risk group of patients were classified according to the National Protocol for the Treatment of Childhood Cancer [31], modified from the INRG staging system (INRGSS), and reviewed and decided by pediatric oncologists.

Informed consent for the use of human samples was obtained from all patients. This research was conducted in accordance with the Declaration of Helsinki. Ethical approval was granted by the Human Research Ethics Committee of the Faculty of Medicine, Prince of Songkla University, and Mahidol University under certificates of approval REC.64-195-10-1 and COA. MURA2021/661, respectively.

### 2.2. DNA Extraction and Whole-Exome Sequencing

Genomic DNA was isolated from freshly frozen tumor samples using the DNeasy Blood & Tissue Kit (Qiagen, Hilden, Germany) following the manufacturer’s instructions. DNA concentration and purity were measured using a NanoDrop spectrophotometer (Thermo Fisher Scientific, Dreieich, Germany), and DNA integrity was assessed using an Agilent 2200 TapeStation System (Agilent Technologies, Santa Clara, CA, USA). Exome sequencing libraries were generated using Agilent SureSelect Human All Exon v6 or Agilent SureSelect Human All Exon v7 kits (Agilent Technologies, Santa Clara, CA, USA) following the manufacturer’s protocol. The DNA libraries were quantified using the Qubit dsDNA HS assay kit (Thermo Fisher Scientific, Dreieich, Germany), and their sizes were determined using the 4150 TapeStation system with an Agilent D1000 ScreenTape assay (Agilent Technologies, Santa Clara, CA, USA). The captured DNA fragments were sent to Macrogen for sequencing on an Illumina NovaSeq 600 (Macrogen, Seoul, Korea) with an expected average coverage of 100×, generating 150 bp paired-end reads per sample.

### 2.3. Variant Calling and Annotation

After sequencing, the raw FASTQ files underwent quality and adapter content contamination checks using FastQC version 0.12.1 [32]. Illumina adapters, low-quality bases, and short reads (<36 bp) were removed using Trimmomatic version 0.39 [33]. The trimmed reads were then subjected to another quality check using Fast QC. Subsequently, the resulting high-quality reads were preprocessed following GATK Best Practices Workflows for variant discovery to produce analysis-ready BAM files [34]. These reads were aligned with the human reference genome GRCh38 (hg38) from the GATK resource bundle using the Burrows–Wheeler aligner-MEM (BWA 0.7.17) [35]. The resulting SAM (Sequence Alignment/Map) files were converted into BAM (Binary Alignment/Map) files using Samtools version 1.9 [36]. Duplicate marking and sorting were performed using MarkDuplicatesSpark [37]. To improve insertion/deletion (indel) detection sensitivity, we executed the indel realignment step using ABRA2 [38]. The statistics of the aligned sequencing reads, including coverage, were computed using Qualimap v2.3 [39]. Following base quality score recalibration, somatic variants were called using the tumor-only mode of Mutect2 [40]. This mode was run using the 1000 Genome Project panel of normals [41] and gnomAD (Genome Aggregation Database) germline resource [42] obtained from the GATK resource bundle. According to the GATK Best Practices for somatic short variant discovery, we filtered out cross-sample contamination, single-stranded substitution errors, polymerase slippage artifacts, and germline variants called somatic variants using GATK’s GetPileupSummaries, CalculateContamination, LearnReadOrientationModel, and FilterMutectCalls [43]. Somatic variants with high confidence were annotated using ANNOVAR [44].

### 2.4. Variant Refinement

All somatic variants fulfilling the following criteria [45] were retained for downstream analysis: (i) a ‘PASS’ filter, (ii) read depth ≥ 20, (iii) variant allele frequency (VAF) ≥ 5%, (iv) exonic or splicing variants based on RefGene from the National Center for Biotechnology Information, (v) nonsynonymous variants (excluding synonymous and unknown variants), (vi) variants present in our customized cancer-associated gene panel (1132 genes, Supplementary Table S1) derived from the curated literature and public resources, including the Cancer Gene Consensus of COSMIC v98 database for GRCh38 and the Pediatric Cancer Variant Pathogenicity Information Exchange [23,46,47,48,49,50,51], (vii) minor allele frequency (MAF) ˂ 0.2% in any global or subpopulation dataset of Exome Sequencing Project 6500, Exome Aggregation Consortium, and 1000 Genomes [45], (viii) variants not classified as ‘benign’ or ‘likely benign’ in the ClinVar annotation.

### 2.5. Data Analysis and Visualization of Somatic Mutations

The R bioconductor package “maftools” (version 2.16.0) was employed to analyze and visualize the filtered and annotated somatic variants [52]. Summary plots, oncoplots, and VAF plots were generated using maftools. Lollipop plots were used to depict the genomic loci of amino acid changes for frequently mutated genes. The enrichment of all mutated genes in known oncogenic signaling pathways from The Cancer Genome Atlas cohort (i.e., Cell Cycle, Hippo, MYC, NOTCH, NRF2, PI3K, TGF-Beta, RTK-RAS, TP53, and WNT) was examined and visualized using the R package maftools. The somatic interactions among a set of candidate genes containing mutually exclusive or co-occurring mutations were evaluated. A pairwise Fisher’s exact test was performed to detect significant gene pairs (*p*-value < 0.05) among mutated genes.

### 2.6. Tumor Mutational Burden

Tumor mutational burden (TMB) was estimated based on the total number of somatic nonsynonymous variants divided by the total size of targeted coding regions in million base pairs (Mb) and reported as mutations per Mb. These nonsynonymous mutations included single-nucleotide variants, splice-site variants and short indels. Single-nucleotide variants (SNVs) and small indels were also identified. Thus, the exonic variants satisfying the aforementioned criteria, excluding IV (only splicing variants were not included), VI, and VIII, were used to calculate the TMB in each sample using maftools. In this context, we further divided the patients into high and low TMB groups based on the median TMB levels. We used the non-parametric Kruskal–Wallis test to examine the correlation between TMB values and clinicopathological parameters.

### 2.7. Mutational Signature Analysis

We used Mutalisk [53] to determine the mutational signatures of all high-confidence somatic SNVs that were not filtered using our validated custom gene panel and the ClinVar annotation criteria. The GRCh38/hg38 (*Homo sapiens*, humans) genome assembly was selected before uploading a VCF file for each sample. The mutational spectrum was decomposed using 30 known single base substitution (SBS) signatures v3.4 in the Catalogue of Somatic Mutations in Cancer (COSMIC) database [50]. The relative contribution of each mutational signature was calculated using linear regression. The best decomposition model is automatically proposed based on the Bayesian information criterion. A heat map representing the relative contribution of each mutational signature in each sample was generated using the Morpheus online tool from the Broad Institute (https://software.broadinstitute.org/morpheus/, accessed on 5 March 2024).

### 2.8. Survival Analysis

We defined OS as the period between the time of diagnosis and death from any cause or the last follow-up. Kaplan–Meier curves were used to estimate the OS distributions of patients with high-frequency gene mutations. Survival curves were compared using the log-rank test, and *p*-values were calculated using GraphPad Prism version 8.0.1 for Windows (GraphPad Software, San Diego, CA, USA). A statistically significant difference was defined as a log-rank test *p*-value < 0.05.

### 2.9. Cancer Driver Gene Identification

Quality-filtered somatic alterations in MAF files were uploaded to the Cancer Genome Interpreter (CGI) online platform [54] to assess their potential as tumor driver mutations and their relevance to possible anticancer therapies and drug responses. NB was selected as the cancer type for the CGI annotation.

### 2.10. Identification of Chemoresistance Markers

We divided patients with NB into two groups according to their tumor response as defined by the RECIST criteria [55]. Chemotherapy sensitivity was defined as complete or partial response, while stable or progressive disease was classified as chemotherapy resistance. The proportion of patients with specific gene mutations between the two groups was compared using Fisher’s exact test, and a *p*-value < 0.05 was considered statistically significant.

### 2.11. GO and Pathway Enrichment Analysis

To elucidate the enriched GO terms and pathways of the mutated genes, we conducted an overrepresentation analysis using the Database for Annotation, Visualization, and Integrated Discovery web-based tool [56]. GO functional annotation included the categories of biological processes, cellular components, and molecular functions. Statistical significance for both the GO function and KEGG pathways was considered when the false discovery rate-corrected *p*-value was less than 0.05.

### 2.12. PPI Network and Identification of Hub Genes

We used the Search Tool for the Retrieval of Interacting Genes (STRING) database (https://string-db.org/; version 12.0, accessed on 4 March 2024) to construct an interaction network among the proteins encoded by all mutated genes. A minimum interaction score of 0.400 (medium confidence) was set as the threshold for visually analyzing associations between mutated genes. Disconnected nodes in the network were excluded. The STRING network was then imported into the Cytoscape software (version 3.10.1) for better visualization and further analysis [57]. The CytoHubba plugin [58] was used to identify the top ten core genes using the Maximum Clique Centrality (MCC) algorithm.

### 2.13. Statistical Analysis

Statistical analyses were performed using IBM SPSS Statistics 26 software for Windows (IBM, Armonk, NY, USA). Fisher’s exact test was used to compare categorical variables. Given the non-normal distribution of variables, the Mann–Whitney U-test was used for comparisons between the two groups. A *p*-value < 0.05 was considered statistically significant.

## 3. Results

### 3.1. Patient and Tumor Characteristics

A total of 48 tumor samples from patients diagnosed with NB were included in this study. The baseline and clinicopathological features of the participants and samples from our cohort are detailed in Table 1. Among the patients, 32 (66.7%) were male and 16 (33.3%) were female, with a median age at diagnosis of 2.7 years (range 0.3–16.8 years). The majority fell into the age group of ≥18 months (36/48; 75%). A total of 43 patients (89.6%) were classified as INRG stage M and INSS stage 4, and 44 patients (91.7% of the 48 enrolled) were categorized into the high-risk group according to the National Protocol for the Treatment of Childhood Cancer [31]. As expected, most NB tumors were located in the adrenal glands (26/48, 54.2%). NB was the most common type of malignant tumor (30/48, 62.5%), followed by ganglioneuroblastoma (9/48, 18.8%) and ganglioneuroma (6/48, 12.5%). Metastasis occurred at various sites in 46 patients (95.8%), predominantly in the bone (38/45, 84.4%) and bone marrow (26/45, 57.8%). Recurrence was observed in only four cases (8.3%). Of the 48 patients enrolled in the study, 43 (89.6%) had received chemotherapy for NB, and 26 (54.2%) showed a complete response. Radiation therapy was administered to some patients. Patients received radiation therapy for their tumors with the following total radiation doses: 21 Gy in 14 fractions for two patients, 20 Gy in 10 fractions for one patient, 25.5 Gy in 17–20 fractions for one patient, and 30 Gy in 10 fractions for one patient. The duration of radiation therapy varied among patients. Forty patients (83.3%) were alive at the last follow-up, while seventeen patients (35.4%) were alive at 5 years.

### 3.2. Whole-Exome Sequencing Statistics

Whole-exome sequencing was performed on DNA extracted from 48 fresh-frozen tumor-only samples. The sequencing and mapping metrics of the indel-realigned bam files were calculated using Qualimap software (v.2.2.2). Each tumor sample had an average of 5,676,815,827.75 high-quality bases, with 99.96–99.98% of sequence reads mapped to the coding regions of the human reference genome. The coverage depth of whole exomes ranged from 62.68× to 105.09×, and the mean coverage depth of 92.69× was obtained when considering all tumor exome sequences.

### 3.3. Mutation Landscape of Patients with NB

**Figure 1:**
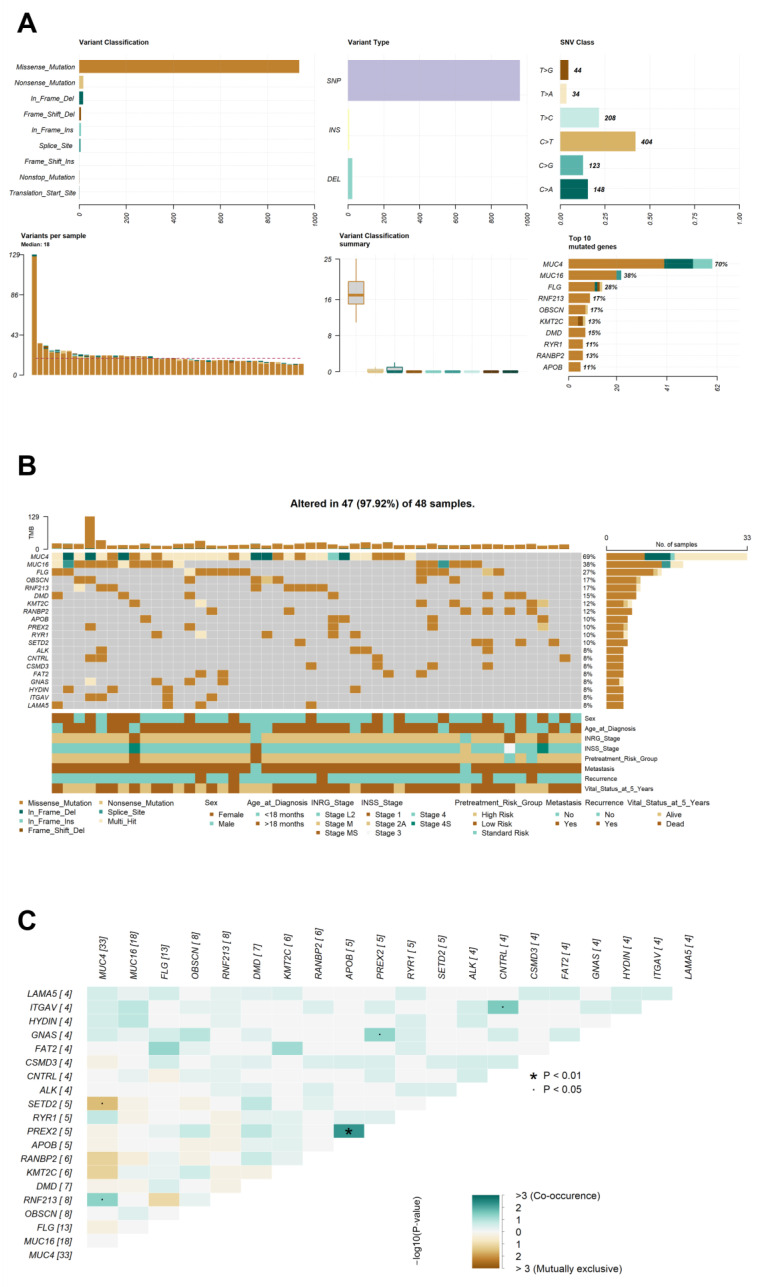
Landscape of somatic variants in patients with NB. (**A**) The mutation summary in NB displays the distribution of variant classification, variant type distribution, base substitution type, the number of somatic variants in each sample, a summary of variant classification, and the top 10 genes with the highest number of mutations. (**B**) Oncoplot of the top 20 mutated genes in 48 NB cases. (**C**) Co-occurring and mutually exclusive interactions among the top 20 frequently mutated genes. A tendency toward co-occurrence and mutual exclusivity is represented with bluish-green and brown, respectively. The statistical significance for each pair of genes is determined using Fisher’s exact test.

To explore the somatic mutation profiles of patients with NB, we used the “maftools” package in the R program to analyze and visualize somatic mutation data. The details of the somatic mutations in both the exon and splice sites of all the mutated genes are summarized in Figure 1A and Supplementary Table S2. After filtering the variants using our stringent criteria, we identified 995 somatic mutations in 47 samples (97.9%), affecting 572 cancer-associated genes. The VAF of the top 10 mutated genes are shown in Supplementary Figure S1. The resulting VAF demonstrated that both *MUC4* and *KMT2C* were clearly separated from the other genes, indicating their non-clonal status [59]. The overall variant classification and SNV class are presented as bar plots of various colors. Regarding variant classification, missense mutations were the predominant type, followed by nonsense mutations and in-frame deletions. In each variant classification, there were 935 missense, 18 nonsense, 17 in-frame deletions, 8 in-frame insertions, 8 frameshift deletions, 6 splice-site mutations, 1 frameshift insertion, 1 nonstop, and 1 translational start-site mutation.

Single-nucleotide variants (SNVs) comprised most variant types (961 variants), whereas deletions (25 variants) and insertions (9 variants) were less common. Among these, 955 SNVs and 34 indels were identified in exonic regions. The SNV classes C > T and T > C were dominant, with transition mutations being more common than transversions. The number of somatic variants in each sample ranged from 12 to 129, with a median of 18. The number of different variant classifications for each sample is shown as a box plot. An oncoplot was generated to display the comprehensive somatic mutation profiles of all samples in our NB cohort, listing the 20 genes with the highest mutation rates as percentages (Figure 1B). The ten most frequently mutated genes were *MUC4* (70%), *MUC16* (38%), *FLG* (28%), *OBSCN* (17%), *RNF213* (17%), *DMD* (15%), *KMT2C* (13%), Ran-binding protein 2 (*RANBP2)* (13%), apolipoprotein B (*APOB)* (11%), and *PREX2* (11%). A total of 187 recurrently mutated genes (>two patient samples) were identified; however, no common somatic variants were detected in any of the NB tumor specimens.

### 3.4. Detection of Co-Occurring Mutated Genes

Next, we investigated whether any of the mutated genes identified in patients with NB co-occurred or were mutually exclusive. Somatic interactions were analyzed using maftools, and events among the top 20 mutated genes are shown in Figure 1C, where green and brown represent co-occurrence and mutually exclusive events, respectively. *MUC4* mutations were significantly correlated with *SETD2* in a mutually exclusive manner (*p* < 0.05). Regarding co-occurrence relationships, we found that *APOB* and *PREX2* co-existed, which was statistically significant (*p* < 0.01). Furthermore, somatic variants in *MUC4*, *PREX2*, and *CNTRL* had a co-occurrence relationship with *RNF213*, *GNAS*, and *ITGAV* mutations, respectively (*p* < 0.05).

### 3.5. Distribution of Mutations in the Top Six Mutated Genes

**Figure 2:**
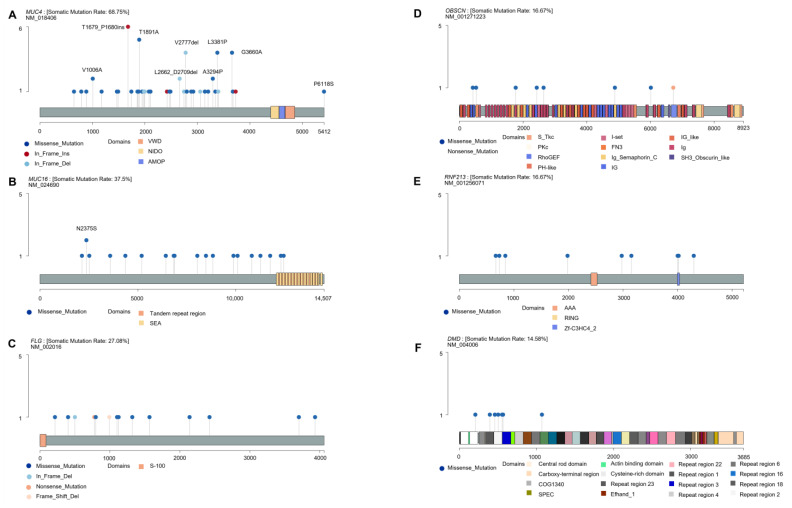
The lollipop plot of the top six most commonly mutated genes. (**A**) *MUC4*. (**B**) *MUC16*. (**C**) *FLG*. (**D**) *OBSCN*. (**E**) *RNF213*. (**F**) *DMD*. The x-axis represents the mutation position relative to a schematic representation of a gene and the y-axis represents the total number of mutations at each alteration.

The lollipop plot was generated using maftools to examine mutation hotspots in specific functional domains and identify recurrent mutations in the six most frequently mutated genes (Figure 2A–F). Overall, 60 somatic mutations were detected in *MUC4* (12 deletions, 8 insertions, and 40 SNPs), 22 in *MUC16* (22 SNPs), 14 in *FLG* (2 deletions and 12 SNPs), 8 in *OBSCN* (8 SNPs), 9 in *RNF213* (9 SNPs), and 7 in *DMD* (7 SNPs). Protein alterations in at least 2 samples were identified only in *MUC4* (33 samples) and *MUC16* (18 samples), which were ranked among the top two mutated genes. All mutations in *MUC4* were located in exon 2, except for one alteration, p.E788K, found in exon 17 in a single sample (Figure 2A). Additionally, no mutations were observed in the functional domain of *MUC4*. The most frequently detected alteration was the insertion of p.T1679_P1680insSLPVTSTSSASTGHATPLPVTDNSSVSTGHAT (six samples), followed by the variant p.T1891A (five samples). We identified eight recurrent mutations: p.T1679_P1680insSLPVTSTSSASTGHATPLPVTDNSSVSTGHAT, p.T1891A, p.V2777del, p.L3381P, p.G3660A, p.V1006A, p.L2662_D2709del, and p.A3294P. The alteration p.P6118S was located on an alternate contig of GRCh38, which was outside the length of *MUC4* transcription (NM_018406). For the *MUC16* gene, we also observed a recurrent variant affecting the asparagine residue at amino acid 2375 (p.N2375S) and identified two missense alterations in the SEA domain (Figure 2B). Multiple mutations were found exclusively in exon 3 of *FLG* (Figure 2C). Among the eight variants identified in *OBSCN*, seven were missense variants and one was a nonsense variant (Figure 2D). These mutations occurred at multiple locations in exons 4 (p.L423H), 5 (p.S528Y), 18 (p.D1772Y), 27 (p.T2432M), 29 (p.G2646S), 55 (p.Y4886H), 75 (p.E6017D), and 92 (p.W6720X). Two missense variants (p.G4002E and p.L4021F) in the zf-C3HC4_2 domain were observed in *RNF213* (Figure 2E). *DMD* possessed all missense mutations that were biased toward the repeat regions (Figure 2F).

### 3.6. Correlation of Mutational Status of Frequently Mutated Genes with Clinicopathological Parameters and Their Prognostic Impact

We also investigated the association between mutations in the top 10 most mutated genes and clinicopathological variables (Supplementary Table S3). No significant differences in age at diagnosis were observed between patients with wild-type and mutated *MUC4*, *MUC16*, *FLG*, *OBSCN*, *RNF213*, *DMD*, *KMT2C*, *RANBP2*, *APOB*, or *PREX2*. Additionally, results from the Fisher’s exact test indicated no significant association between these two groups with respect to the evaluated clinicopathological parameters. However, we found that the mutational status of *DMD* was significantly associated with patient sex (*p* = 0.033). The *RNF213* wild type was significantly correlated with bone metastasis (*p* = 0.047). Furthermore, *RANBP2* mutations correlated with the pre-treatment risk group (*p* = 0.027), whereas *APOB* mutations were associated with the response to treatment (*p* = 0.021).

**Figure 3:**
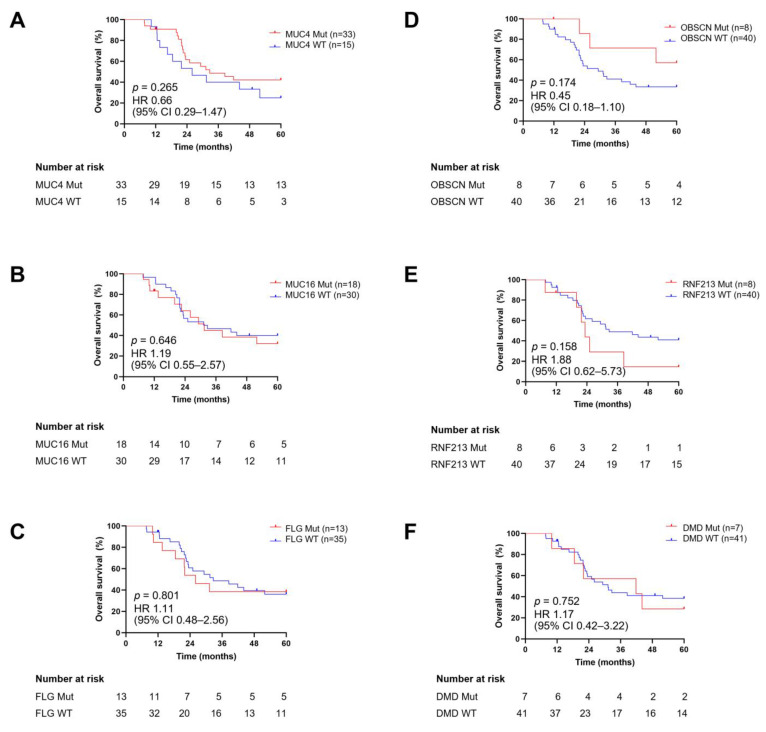
Correlation between somatic mutational status and overall survival (OS) of patients with NB. (**A**) Wild-type and mutated *MUC4*. (**B**) Wild-type and mutated *MUC16*. (**C**) Wild-type and mutated *FLG*. (**D**) Wild-type and mutated *OBSCN*. (**E**) Wild-type and mutated *RNF213*. (**F**) Wild-type and mutated *DMD*. Censored subjects are denoted with tick marks on the curves. The log-rank test was used to compare differences in OS between patients with wild-type genes and mutations.

We assessed the prognostic value of the six frequently mutated genes through survival analysis using Kaplan–Meier curves (Figure 3A–F). The mutational status of these genes was categorized as wild-type or mutated. The follow-up duration for patients with NB ranged from 7.66 to 60 months, with a median of 30.28 months. Our results demonstrated that the OS of patients with mutations in their tumors for *MUC4*, *MUC16*, *FLG*, *OBSCN*, *RNF213*, and *DMD*, as well as those without mutations, did not show significant differences (*p* = 0.265, 0.646, 0.801, 0.174, 0.158, and 0.752, respectively).

**Figure 4:**
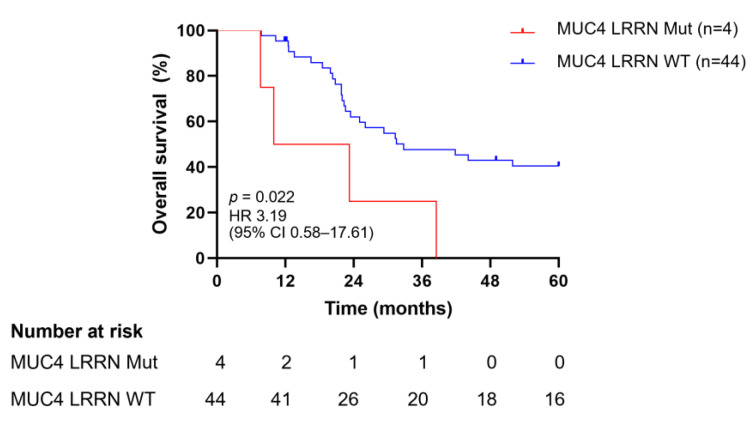
Correlation between mutated *MUC4 LRRN* and OS of patients with NB. Censored subjects are denoted with tick marks on the curves. The log-rank test was used to compare differences in OS between patients with wild-type genes and mutations.

We further evaluated the association between combinations of two mutated genes and OS. Mutations in both *MUC4* and *LRRN3* were negatively correlated with survival (hazard ratio [HR]: 3.19, 95% CI: 0.58–17.61) (Figure 4), while other combinations were not significantly associated when compared with the wild type. These mutations significantly decreased the median survival time to 16.62 months.

### 3.7. TMB Distribution and Its Association with Age, Sex, Primary Site of Tumors, and Survival Outcomes

**Figure 5:**
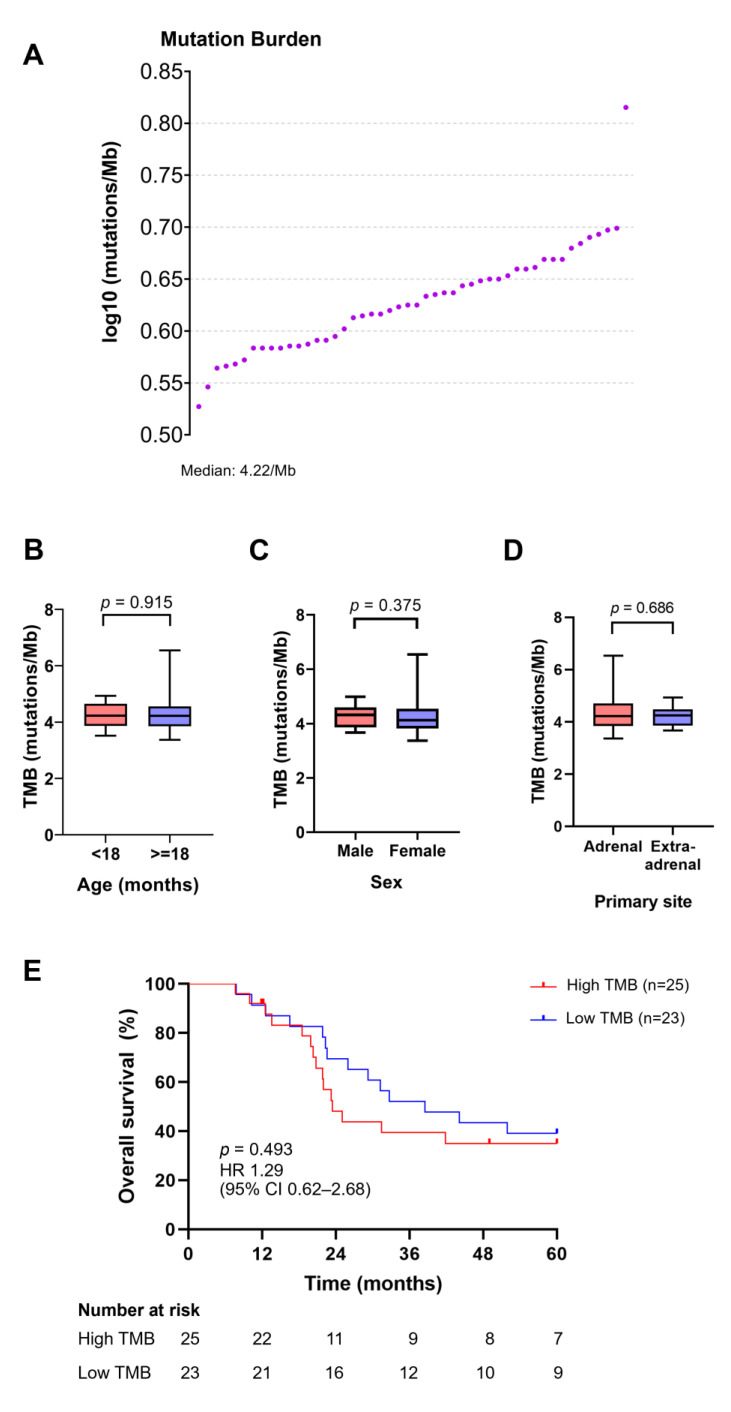
Tumor mutational burden (TMB), its prognostic value and association with clinicopathological characteristics, and comparison of TMB. (**A**) TMB scores of each patient in the NB cohort. (**B**–**D**) Correlation analysis between TMB and clinicopathological factors, including age, gender, and primary site. (**E**) Survival analysis of patients with high and low TMB.

The TMB value in our cohort, expressed as the total number of mutations per million bases, varied from 3.37 to 6.54 mutations/Mb, with a median TMB value of 4.22 mutations/Mb (sA). Based on the median TMB, 25 patients were classified into the high TMB group (≥4.22 mutations/Mb), while the rest were in the low TMB group (<4.22 mutations/Mb). We then explored the differences in TMB levels among patients with NB of different age groups, sexes, and primary tumor sites. There were no significant differences in the TMB values associated with these clinicopathological parameters (Figure 5C–E). Similarly, the survival outcomes between patients with high and low TMB were not significantly different (*p* = 0.493).

### 3.8. Mutational Signature of NB

**Figure 6:**
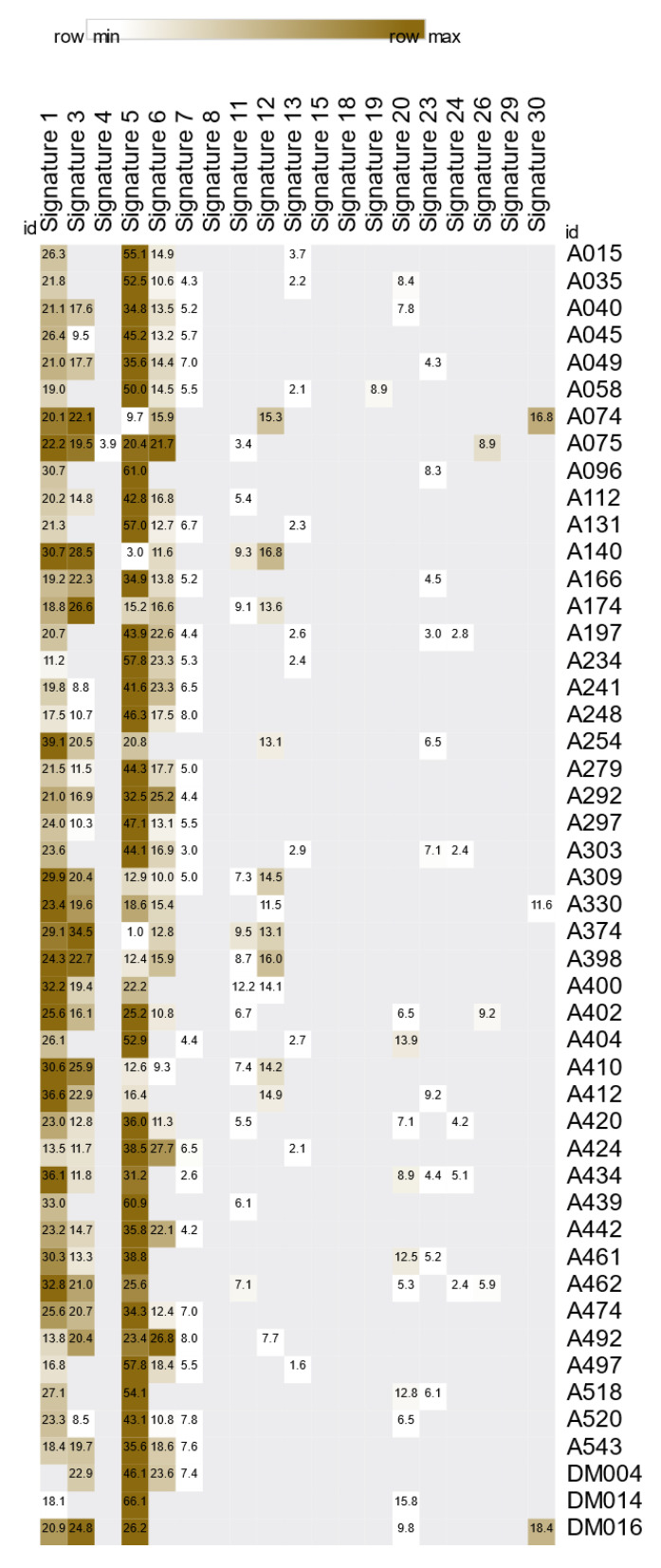
Mutational signatures enriched in the NB cohort. Columns represent the COSMIC signatures and rows indicate the samples. The numbers in the matrix represent the percentage of the relative contribution of signatures, with darker brown indicating a higher percentage contribution of the COSMIC signature.

To examine the mutational processes resulting in somatic alterations in the NB cohort, signature decomposition was performed on datasets based on 30 COSMIC mutational signatures. The relative contribution as a percentage for each signature was calculated and plotted as a heat map in Figure 6. Our NB cohort exhibited various signatures including SBS1, SBS3, SBS4, SBS5, SBS6, SBS7, SBS11, SBS12, SBS13, SBS19, SBS20, SBS23, SBS24, SBS26, and SBS30. Five of these signatures (3, 6, 20, 26, and 30) were linked to the mutational processes underlying defective DNA repair. The C > T transition was the most evident, with relative contributions ranging from 39.3% to 49.1%. Furthermore, T > C had the second highest proportion of SBS (Supplementary Figure S2). The COSMIC signature 5 was observed in all samples, with 32 of them exhibiting the highest contribution (≥26%) compared to the other SBS signatures. This signature followed a clock-like pattern, indicating that the number of mutations was related to patient age. Subsequently, signature SBS1 was enriched in 97.9% of the samples, and its etiology stemmed from the spontaneous or enzymatic deamination of 5-methylcytosine to thymine (another clock-like signature). Signatures 3 and 6, corresponding to deficiencies in DNA repair mechanisms (homologous recombination-based DNA damage repair for 3 and DNA mismatch repair for 6) were found in 35 (72.9%) and 36 (75.0%) of the 48 samples, respectively. In summary, the most common mutational signature in the NB cohort was SBS5 (48/48, 100%), followed by SBS1 (47/48, 97.9%), SBS6 (36/48, 75.0%), and SBS3 (35/48, 72.9%).

### 3.9. Enrichment of Mutated Genes in Oncogenic Signaling Pathways

**Figure 7:**
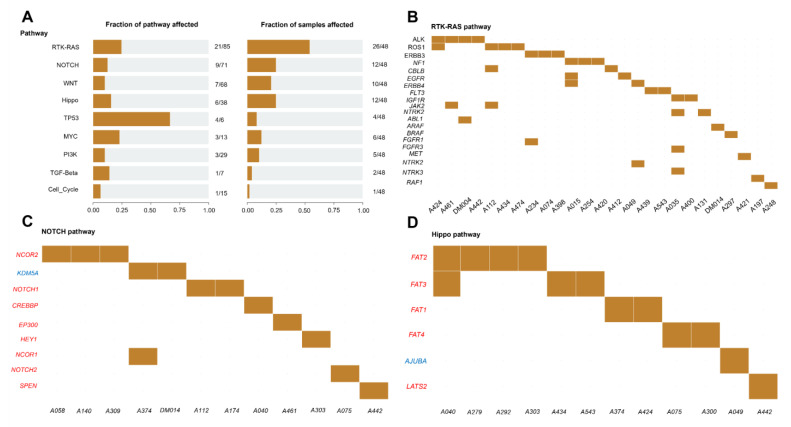
Enriched oncogenic signaling pathways in NB. (**A**) Oncogenic pathways based on the number of mutated genes in the pathway and samples. (**B**–**D**) Somatic variants in genes of the RTK-RAS, NOTCH, and Hippo pathways across different samples. Blue text represents oncogenes, and red text represents tumor suppressor genes.

To explore the oncogenic signaling pathways involving all mutated genes, we conducted a pathway analysis using the R package maftools (Figure 7A–D). The results revealed the enrichment of mutated genes in several oncogenic pathways: TP53 (4/6 genes, 66.7%), RTK-RAS (21/85 genes, 24.7%), MYC (3/13 genes, 23.1%), Hippo (6/38 genes, 15.8%), TGF-Beta (1/7 genes, 14.3%), NOTCH (9/71 genes, 12.7%), WNT (7/68 genes, 10.3%), PI3K (3/29 genes, 10.3%), and cell cycle (1/15 genes, 6.7%). Considering the number of samples, the most disrupted oncogenic pathways were RTK-RAS (26/47 samples, 55.3%), NOTCH (12/47 samples, 25.5%), Hippo (12/47 samples, 25.5%), WNT (10/47 samples, 21.3%), MYC (6/47 samples, 12.8%), PI3K (5/47 samples, 10.6%), TP53 (4/47 samples, 8.5%), TGF-Beta (2/47 samples, 4.3%), and cell cycle (1/47 samples, 2.1%) (Figure 7A). The mutated genes associated with each signaling pathway are listed (Figure 7B–D). The most frequently mutated genes were *ALK* and *ROS1* (4/26 samples, 15.4%) for RTK-RAS (Figure 7B), *NCOR2* (3/12 samples, 25%) for NOTCH (Figure 7C), *APC* (3/10 samples, 30%) for WNT, *FAT2* (4/12 samples, 33.3%) for Hippo (Figure 7D), *ATM* (2/4 samples, 50%) for TP53, *MGA* (4/6 samples, 66.7%) for MYC, and *TSC1* for PI3K (3/5 samples, 60%).

### 3.10. Functional Enrichment Analysis of Mutated Genes in NB

**Figure 8:**
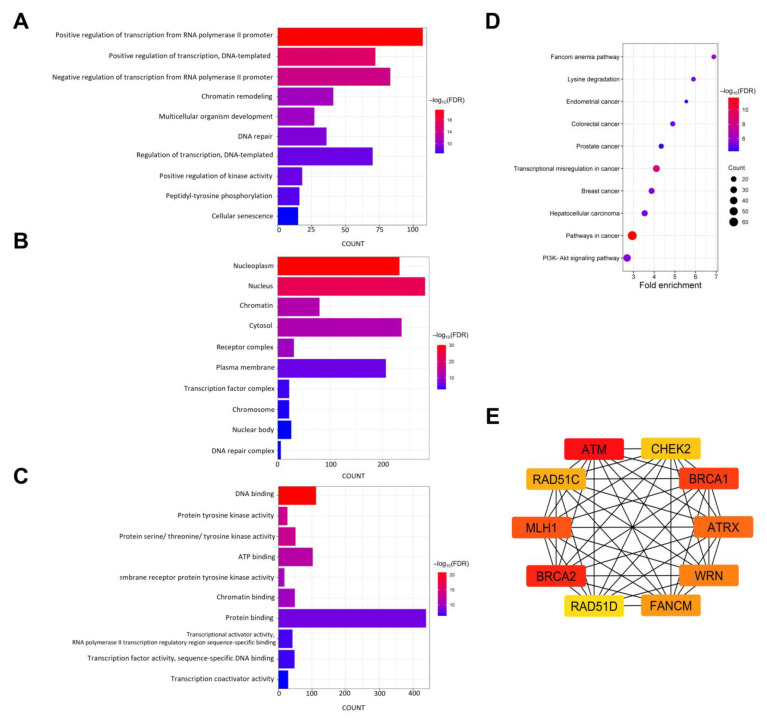
Enrichment analysis of mutated genes in NB. (**A**–**C**) Top 10 significantly enriched Gene Ontology terms in biological process, cellular component, and molecular function. (**D**) Top 10 significantly enriched KEGG pathways. (**E**) Top 10 hub genes. The color gradient from yellow to red indicates the degree of hub importance—yellow for less important hub genes and red for the most important hub genes.

To gain a deeper understanding of the biological significance and pathways associated with the 572 mutated genes in NB, we conducted GO and KEGG pathway enrichment analyses (Figure 8A–D). In terms of biological processes, the GO analysis revealed significant enrichment in the regulation of transcription, kinase activity, proteins (peptidyltyrosine phosphorylation), multicellular organism development, DNA repair, and cellular senescence (Figure 8A). Concerning the GO terms in CC, the mutated genes were predominantly localized in the nucleoplasm, nucleus, chromatin, and cytosol (Figure 8B). Molecular-function GO terms indicated that the mutated genes were primarily involved in DNA, ATP, chromatin, and protein binding, as well as in the activity of transcriptional activators, factors, and coactivators (Figure 8C). KEGG pathway analysis showed significant associations between cancer, Fanconi anemia, and signaling pathways, such as PI3K-Akt and Rap1, for all mutated genes (Figure 8D). Additionally, these genes have been implicated in transcriptional misregulation in cancer, lysine degradation, and various cancers, including hepatocellular carcinoma and breast, colorectal, prostate, and endometrial cancers.

### 3.11. PPI Network and Hub Gene Identification

The PPI network of mutated genes was constructed using STRING, resulting in 570 nodes and 5349 edges. The STRING network was then imported into the Cytoscape software (version 3.10.1) for enhanced visualization. Fifty-four nodes that were not connected to the core network were removed, leaving 516 nodes and 5347 edges for analysis. Hub genes were identified using the CytoHubba plugin in Cytoscape and the MCC algorithm. According to the MCC rank, the top 10 candidate hub genes were *ATM*, *BRCA2*, *BRCA1*, *MLH1*, *ATRX*, *WRN*, *FANCM*, *RAD51C*, *CHEK2*, and *RAD51D* (Figure 8E).

### 3.12. Identification of Cancer Driver Genes and Biomarkers for Therapeutic Target and Drug Response

Next, we investigated whether the somatic variants detected in all samples could be annotated or predicted as driver or passenger mutations using computational methods (oncodriveMUT and boostDM) from the CGI. Out of 964 somatic alterations classified by the CGI server, 122 mutations were identified as candidate “driver” mutations present in 87.5% of the samples, while 792 were classified as “passenger” mutations. Additionally, there were 24 mutations classified as “non-protein affecting” (Supplementary Table S4). A total of 15 of 122 driver mutations were well annotated in Oncokb (4 mutations), both Oncokb and CGI (1 mutation), or ClinVar (10 mutations), and these mutations were distributed in *MUC4*, *ALK*, *BRAF*, and *FGFR1*. Predicted driver mutations were located in 87 different genes. Fourteen samples (29.2%) harbored at least one recurrent driver mutation out of five alterations encompassing several identified driver genes, such as *MUC16*, *MUC4*, *ALK*, and catenin delta-1 (*CTNND1)* (Table 2). We also predicted the deleterious functional effects of recurrent cancer driver mutations using in silico prediction tools from ANNOVAR (Supplementary Table S2). None of the prediction tools appeared to work for analyzing two recurrent variants in *MUC4*. For the *MUC16* gene, MutationTaster and BayesDel (addAF and noAF) classified a recurrent splice-site variant as disease-causing and damaging, respectively. The *ALK* F1174L mutation was predicted to have detrimental effects by SIFT, MutationTaster, PROVEAN, M-CAP, and BayesDel (addAF and noAF). Similarly, somatic driver variants in the *CTNND1* gene were predicted to negatively affect protein function by SIFT, MutationTaster, PROVEAN, and M-CAP. Concerning recurrent driver mutations, splice-site driver variants in *MUC16* were present in two samples (4.2%). The *MUC4* gene harbored in-frame insertions of 32 nucleotides between threonine at position 1679 and proline at position 1680 (p.T1679_P1680ins). Concerning the *ALK* gene, we observed the recurrent mutation F1174L along with *ALK* R1275Q in a single sample. Remarkably, the recurrent missense mutation R439C in the *CTNND1* gene was also discovered. This novel driver mutation was identified in two tumor samples and further validated by Sanger sequencing using the Applied Biosystems 3730/3730xl DNA Analyzer (Supplementary Figure S3). The presence of recurrent cancer driver genes in the mutated samples is summarized in Table 3. In summary, the average number of driver mutations per sample was 2.5, with a median of 2.5 (mutation range, 0–6). According to the list of the top 10 most frequently mutated genes, *MUC4*, *MUC16*, *KMT2C*, and *PREX2* were annotated as cancer driver genes. Among the 15 recurrent driver genes, *MUC4* was the most prevalent in the samples (12/48, 25.0%), followed by *KMT2C* (4/48, 8.3%) and *PREX2* (4/48, 8.3%). The remaining tumors were detected in two tumor samples.

For the CGI annotation in personalized therapy (Supplementary Table S5), we selected only druggable alterations that completely matched the amino acid alterations identified in the same genes and tumor types (neuroblastoma or any cancer type) as the observed biomarkers. Ten actionable mutations were identified in eleven samples (22.9%) spanning seven genes: *NF1*, *ALK*, *SETD2*, *BRCA1*, *NOTCH1*, *ATR*, and *FGFR1* (Table 4). These biomarkers are supported by evidence from early trials (four mutations in *ALK*, *BRCA1*, and *NOTCH1*) and pre-clinical data (eight mutations in *NF1*, *ALK*, *SETD2*, *ATR*, and *FGFR1*). Among them, two samples harbored *ALK* F1174L mutations as potential drug targets and were responsive to alectinib, crizotinib, TAE684, AZD3463, and lorlatinib. However, this mutation may confer crizotinib resistance. The R1275Q mutation in *ALK* was also identified as a therapeutic target for crizotinib, lorlatinib, and TAE684 in one of our tumor samples, although this mutation was likely to exhibit resistance to TAE684. Nevertheless, supporting evidence for such treatment is in the clinical trial stage. We identified three *NF1* oncogenic mutations (Q1798*, Q2616*, and S636X) that may confer resistance to retinoic acid. The predicted driver mutations responsive to WEE1 inhibitors included P10L and Q1829E for *SETD2* and R612S for *BRCA1*. We also analyzed these actionable variants in other tumor types for drug repurposing. In this context, we identified 24 predictive biomarkers for drugs used to treat various cancers. Several drugs are available in the guidelines or in late-stage trials.

### 3.13. Identification of Biomarkers Associated with Chemoresistance

**Figure 9:**
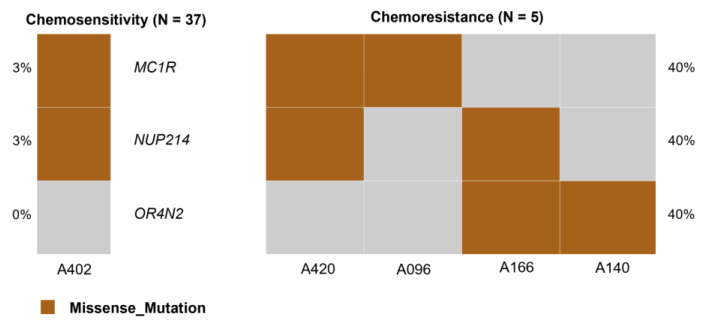
An oncoplot of somatic mutations associated with chemotherapy resistance in patients from both chemosensitivity and chemoresistance groups.

To identify predictive markers of chemoresistance, we compared the proportion of somatic mutations in the chemosensitivity and chemoresistance groups using Fisher’s exact test. The results showed that variants in *OR4N2*, *MC1R*, and *NUP214* were significantly associated with chemotherapy resistance (*p*-value < 0.05) (Figure 9). The CGI results revealed 14 cancer driver mutations in 12 genes: *EPHA2*, *NSD1*, *BUB1B*, *CTNND1*, *KAT6A*, *MUTYH*, *MAP3K13*, *MUC4*, *KMT2C*, *VAV1*, *BIRC6*, *NF1*, and *MUC4* (Supplementary Table S4). Each patient with chemoresistance harbored at least one driver mutation, and no recurrent drivers were identified. The CGI provided a list of specific drugs with their responses in patients who had these alterations that might serve as alternative therapeutic options (Supplementary Table S5). One out of four patients with the S636 residue of the *NF1* gene had favorable responses with cobimetinib and trametinib. These drugs are effective in any cancer type and have been reported in pre-clinical studies. The EGFR mAb inhibitors, namely cetuximab and panitumumab, may be the pertinent choice in combating chemotherapy-resistant NB, as all patients in the chemoresistance group had wild-type *KRAS*, indicating responsiveness. Both cetuximab and panitumumab have demonstrated their efficacy against colorectal adenocarcinoma and have been evidenced in guideline-level studies.

## 4. Discussion

NB is an aggressive extracranial malignant tumor in young children that manifests a high degree of clinical and biological heterogeneity, posing a major challenge for pediatric treatment, specifically chemotherapy. For those classified into the high-risk group according to the revised COG criteria, the 5-year OS probability fell below 50% despite the introduction of intensive multimodal therapies. Furthermore, high-risk patients with NB may not respond to chemotherapeutic drugs after prolonged treatment, resulting in poor prognosis and clinical outcomes. Given the extreme variations in NB tumors and the need to develop treatment strategies for patients with high-risk NB, the adoption of personalized medicine as a new and promising approach to tackle this cancer is in demand. With the advent of high-throughput sequencing technologies, it is currently feasible to obtain massive amounts of genetic information from tumors to facilitate tailoring therapy for each patient, leading to further favorable outcomes and minimal toxicity. Nevertheless, the comprehensive genetic alterations underlying NB, especially in high-risk cases, are poorly understood, hampering the development of targeted therapies. In this study, we attempted to unveil the distinct mutational landscape of freshly frozen tumor samples from 48 Thai patients with NB, particularly in the high-risk group. Despite the limited number of samples, we also assessed the tumor mutational burden and explored the correlation between the clinical parameters of patients with NB and frequently mutated genes, as well as TMB. We further sought to identify candidate cancer driver mutations and genes and their implications as putative actionable biomarkers to predict drug responsiveness before genomics-guided cancer treatment. A wide array of bioinformatics tools were used to gain insights into mutational and oncogenic signatures, the functional and pathway enrichment of somatic mutations, and crucial proteins responsible for NB.

In our cohort, the proportion of male patients (66.7%) was higher than that of female patients (33.3%), which is consistent with a previous study from Thailand that reported that 57.9% male and 42.1% female patients had high-risk NB [60]. The incidence of NB in boys has been observed in children in Southern–Eastern Europe [61]. At diagnosis, the median age of the patients in the Southern–Eastern Europe cohort was lower than that in the current study (2 years vs. 2.7 years). The higher median age at diagnosis of patients in this study may partially result from the lack of an established screening program for NB in Thailand, which might delay diagnosis. Another possibility is that most cases were referred from other hospitals, leading to a later date of diagnosis recorded in our hospitals’ medical records. Patients older than 18 months with INRG stage M and INSS stage 4 were predominant, which aligned with the former high-risk neuroblastoma cohort with autologous stem cell transplants [62]. Patients aged more than 18 months with metastatic tumors (stage M) could be classified into a high-risk group according to the National Protocol for the Treatment of Childhood Cancer [31]. The findings of ganglioneuroma and ganglioneuroblastoma histology of some samples are most likely due to the introduction of chemotherapy, as it is well-known that chemotherapy contributes to the maturation of neuroblastomas [63]. This explanation is supported by the observation of neuroblastoma histology in all patients before chemotherapy, while more mature stages of neuroblastic differentiation have been seen in post-chemotherapy patients. The high frequency of metastasis observed in this study may be attributed to the fact that most severe patients are referred to our hospitals, which are tertiary hospitals. Additionally, the smaller sample size and differences in population compared to the surveillance, epidemiology, and end result data, which are based on the U.S. population, may skew this result. In line with a previous study on NB, the most common sites of metastasis were the bone and bone marrow [64]. Different trends in NB recurrence have been reported, where our recurrence rate was markedly decreased (8.3%) compared to an earlier study (30.3%) [65]. The low recurrence rate may result from differences in the timing of recording patients’ recurrence status. We mark this at the time of sample collection rather than at the last follow-up, which might differ from other studies. Furthermore, the majority of patients showed a favorable response to chemotherapy, and some patients with NB were new cases referred from other hospitals. Our recurrence rate is consistent with the vital status of patients at the last follow-up, with more patients alive than deceased.

Next, we explored the correlation between high-frequency mutations and clinicopathological features. Our findings showed that the DMD mutational status was significantly correlated with the sex of patients with NB. *DMD* is a 427 kDa dystrophin protein that supports the connection between the muscle fiber cytoskeleton and the extracellular matrix in the sarcolemma by interacting with different proteins such as sarcoglycan and dystroglycan. Genomic alterations in the *DMD* gene are clinically implicated in Duchenne muscular dystrophy, an X-linked neuromuscular disorder in a group of hereditary muscular dystrophies resulting from a failure in the protein translation of dystrophin [66]. Moreover, somatic mutations in *DMD* are associated with unfavorable survival outcomes in patients with non-myogenic cancer [67]. The mutational status of *RNF213* (ring finger protein 213), encoding a 590 kDa protein that harbors a really interesting new gene (RING) finger and an AAA ATPase domain [68], displayed a relationship with bone metastasis. In a previous study, *RNF213* mutations were identified in metastatic tumors and associated with tumorigenesis in various cancers (e.g., liver, ovarian, and gastric cancers) [68].

The prognostic value of the most commonly mutated genes for OS in patients with NB was also evaluated (Figure 3). Although we did not find any mutations in *MUC4*, *MUC16*, *FLG*, *OBSCN*, *RNF213*, or *DMD* to be associated with OS, we did find a significant negative association between mutations in *MUC4* and *LRRN3* and OS. As a membrane-bound mucin, *MUC4* is ordinarily expressed in the trachea, salivary glands, colon, reproductive tract, and bronchioles, and its aberration leads to tumor growth, metastasis, and chemoresistance [69]. Furthermore, *MUC4* mutations have been implicated in the poor prognosis of colon cancer [70]. Leucine-rich repeat neuronal protein 3 (LRRN3) belongs to the LRRN family and is highly expressed in the brain, testes, and adrenal glands [71]. A poor survival outcome for patients with NB has been associated with a low expression of LRRN3 [72]. The combination of *MUC4* and *LRRN3* mutations might serve as a negative prognostic biomarker in NB, although further validation in larger cohorts is needed to confirm its utility. *MUC16* encodes the largest membrane-bound mucin and, unlike *MUC4*, is expressed on the ocular surface, reproductive and internal organs, and the epithelium of the upper respiratory tract. In contrast to *MUC4*, mutated *MUC16* may serve as a prognostic marker for favorable survival outcomes in gastric cancer [73]. The filament-aggregating protein, or filaggrin, encoded by the *FLG* gene, is an important molecule that plays a functional role in the skin, cervical, and oral mucosa barrier [74]. The presence of *FLG* mutations in patients with bladder urothelial carcinoma is associated with longer OS [75]. The *OBSCN* gene spans over 150 kb with more than 80 exons and encodes obscurin, a member of the family of sarcomeric signaling proteins [76], which functions as a tumor suppressor and exhibits oncogenic properties in breast and ovarian cancers, respectively. Obscurin participates in many signaling pathways, including RAS, GPCR, Wnt, and p75, and likely regulates cancer development and progression, especially in breast cancer [77]. Liu et al. reported that increased OS in patients with colorectal cancer was significantly correlated with alterations in the *OBSCN* gene [78]. The relationship between mutations in *RNF213* and OS has not previously been documented. Previous studies have shown that *DMD* mutations in non-myogenic tumors are associated with poor OS [79]. Overall, these results should be interpreted carefully because many patients were lost to follow-up.

In this retrospective study, WES was performed solely on fresh-frozen tumor tissues obtained from 48 patients without matched normal samples. We applied stringent filtering criteria for the somatic variant-calling of tumor-only samples following a previous study [45]. This optimal filtering approach refines the sensitivity and specificity for the detection of somatic mutations using Mutect2 in the tumor-only mode. The top 10 genes with the highest mutation frequencies in the NB cohort were *MUC4*, *MUC16*, *FLG*, *OBSCN*, *RNF213*, *DMD*, *KMT2C*, *RANBP2*, *APOB*, and *PREX2* (Figure 1). The frequencies of *MUC4*, *MUC16*, and *KMT2C* have also been substantially high in previous studies, implying their roles in neuroblastoma development [80,81]. Moreover, the frequency of *MUC4* and *MUC16* mutations in our study was considerably higher than that reported in a previous study on *MYCN* non-amplified neuroblastoma (70% vs. 26% for *MUC4* and 38% vs. 14% for *MUC16*). *MUC4* has been reported to participate in the progression of ganglioneuromas. This transmembrane mucin prevents cancer cell adhesion to the primary tumor, resulting in tumor metastasis [82]. Accordingly, we speculated that *MUC4* played a critical role in the development of metastases in the patients in our cohort.

Recurrent variants of *MUC4* were not previously reported, including p.T1679_P1680ins (six samples), p.T1891A (five samples), p.V2777del (four samples), p.L3381P (four samples), p.G3660A (four samples), p.V1006A (two samples), p.L2662_D2709del (two samples), and p.A3294P (two samples). All mutations were located in exon 2, which encodes the largest domain and O-glycosylation site [83]. The accumulation of variants in exon 2 of *MUC4* observed in our study is consistent with an earlier study in patients with lung cancer [84]. The *MUC4* variants p.Ser2666Tyr and p.Ser2661Gly have been reported in pediatric patients with ganglioneuromas [85]. Nevertheless, because the total number of somatic *MUC4* variants in our findings were located outside the functional domains, their impact on the functionality of the MUC4 protein might be questioned, as experimental studies to confirm the functional significance of these mutations in *MUC4* have rarely been performed.

*MUC16*, also known as CA125, is a high-molecular-weight glycosylated protein that mediates the development of breast and lung cancers by interacting with Janus kinase [86]. In NB, alterations in *MUC16* are frequently detected in *MYCN* non-amplified patients [81] and are specific to their relapse status [87]. Our findings revealed a single recurrent mutation, N2375S, in exon 1, which belongs to the glycosylated extracellular sequence of *MUC16* [88]. No common *MUC16* recurrent somatic mutations involved in the development of NB or other tumor types have been validated in other studies.

The *FLG* gene (encoding filaggrin, a barrier-related molecule) in our cohort exhibited a higher frequency of mutations than that in previous studies (28% vs. 4%) [89]; however, the function of *FLG* in NB is still unknown. In line with a previous report, we did not observe any recurrent variants of *FLG* implicated in the tumor progression of NB.

The mutations in *OBSCN* were highly detected in high-risk NB (23.3%) and were found in different cancer types. The *OBSCN* gene takes part in cell survival and apoptosis and is considered a potential driver of breast cancer. When mutated, they contribute to protein loss, leading to an increased exposure to DNA damage [90]. Our results are in contrast with those of a previous study on high-risk neuroblastoma [91], as no recurrent mutations in *OBSCN* were observed. However, the details of the recurrent variants have not been disclosed.

*RNF213* is an established susceptibility gene associated with moyamoya disease, and its important role in tumor suppression via modulation of the MAPK/JNK signaling pathway has been reported in glioblastoma [68]. Qin et al. identified *RNF213* as a new prognostic biomarker for OS in NB [92]. There were no recurrent variants in *RNF213* affecting NB development, as identified in the present and previous studies.

*DMD* is a tumor suppressor implicated in the pathogenesis of many tumor types, such as lymphomas, sarcomas, and melanomas. Mutations in the *DMD* gene were previously identified to play a role in the development of NB, specifically somatic deletions, which are involved in the pathogenesis of olfactory NB [66]. No established recurrent mutations in *DMD* were described in NB.

*KMT2C* is a histone lysine methyltransferase that plays a role in DNA repair and genomic instability. Furthermore, *KMT2C* serves as a driver gene that promotes tumor progression in breast cancer [93], and high mutation rates of *KMT2C* have been identified in various cancer types, particularly in NB [80].

RanBP2 is a 350 kDa protein of the nuclear pore complex possessing Ran-binding domains and rich-FG repeats that bind to Ran GTP with high affinity. RanBP2 was identified as a new tumor suppressor in lung cancer and has been shown to play an essential role in cellular processes and protein stabilization, including the RAN protein [94]. Although aberrations of *RANBP2* have been described to be involved in tumorigenesis, they showed no significant association with NB risk [95].

APOB is a central structural protein component of very-low-density lipoproteins (VLDL) synthesized by the liver and plays a key role in lipid transport in the human body [96]. Previous studies have demonstrated the role of APOB in neural crest development [97], suggesting its association with NB development and progression.

PREX2 is a 183 kDa regulatory protein, known as a guanine nucleotide exchange factor, that stimulates the small guanosine triphosphatase Rac and mediates the Akt and Rac signaling pathways [98]. PREX plays an important role in cancer, as the loss of PREX2a hampers the proliferation, migration, and invasion of NB [99].

In summary, the significant co-occurring pairs of genes observed in our NB cohort included *APOB* and *PREX2*, *RNF213* and *LRRN3*, *CNTRL* and *ITGAV*, and *LAMA5* and *LRRN3*, whereas somatic interactions, in terms of mutually exclusive events, were observed for *MUC4* and *SETD2*. Previously, the coincidence of the mutated gene pair *ARID1A–APOB* and the mutual exclusion of the pair *KRAS*–*APOB* were observed in pancreatic ductal adenocarcinoma with significant differences [100]. Genomic alterations in *PREX2* are often accompanied by *RUNX1T1* expression in metastatic solid cancers [101]. Given the roles of *APOB* and *PREX2* described in previous studies [97,99], we postulated that *APOB* mutations may cooperate with *PREX2* mutations to synergistically promote neural crest proliferation and drive NB development. Previous studies have shown that *RNF213* co-mutates with *NF1* in human breast cancer samples. Mutually exclusive mutations in *MUC4* and *MUC6* have been identified in dermatofibrosarcoma protuberans [102]. Additionally, a mutually exclusive trend with respect to clear cell renal cell carcinoma is toward *SETD2*–*BAP1* mutations [103]. Because none of the co-occurring and mutually exclusive mutations found in this study have been published previously, an assessment of these co-altered genes in neuroblastoma is required to identify their unknown functional interactions and explore potential therapeutic targets.

TMB, which includes programmed death-ligand 1 and programmed cell death protein 1, is considered a novel biomarker for predicting the response to immune-checkpoint blockade therapy. Determining TMB is integral to stratifying patients with cancer who are likely to benefit from immunotherapy. Nevertheless, a universally valid cutoff for all cancers to classify patients into high and low TMB has not been established for WES, although a TMB of 10 mutations/Mb, as determined by the FoundationOne CDx assay, has been FDA-approved as the threshold for treating all solid tumors with programmed cell death protein 1 inhibitors. TMB is typically defined as the total number of nonsynonymous mutations per Mb of exonic regions of the interrogated tumor sequence; however, some studies have used all types of mutations to estimate TMB [104]. Because an earlier study showed that TMB obtained from tumor-only sequencing was overestimated compared with matched tumor–normal sequencing [105], we addressed this result by excluding as many nonsomatic variants as possible using the more stringent filtering approaches proposed by Sukhai et al. [45].

In the present study, the TMB value in individual patients ranged from 3.37 to 6.54 mutations/Mb, and the median TMB was 4.22 mutations/Mb (Figure 4). Compared with two previous cohort studies on MYCN non-amplified (0.66 mutations/Mb) [81] and olfactory NB (0.45 mutations/Mb) [106], the median TMB in our cohort was much higher, potentially suggesting a high number of remaining germline variants (false positive) that could not be totally excluded after using our stringent filtering criteria, the use of different workflows or bioinformatic pipelines for TMB calculation, or distinct patient populations. Currently, there is no consensus TMB threshold for dividing patients into high and low TMB that applies across different tumor types. Several approaches have been proposed to determine specific TMB cut-off values: arbitrary methods; median-, tertile-, quartile-, or log-rank-based methods [107]; pathological image-based methods [108]; graphical methods [109]; and clinical study-based methods [107]. However, due to our constraints of small sample size, the availability of hematoxylin and eosin-stained slides of tumor tissue, and the lack of relevant studies and clinical trials on NB, the median TMB value was used as the final threshold, as previously described [110]. Based on a median TMB as a cutoff, 25 patients were stratified into the high TMB group (≥4.22 mutations/Mb), which may respond well to immune-checkpoint blockades. High TMB values have been postulated to reflect cancer neoantigens in tumors that are targeted and eliminated by activated immune cells called T cells [111].

Our findings demonstrated that the TMB-high group was independent of any clinicopathological parameter, and no significant difference in TMB values was observed between patients with NB with a distinct age, sex, or primary site. In contrast, Hwang et al. reported an association between patients aged 18 months and high TMB levels in patients with NB. We also found that OS did not differ between patients with high and low TMB, although a trend toward poor OS was observed in the high TMB group. Consistently, the 5-year OS in the high TMB group of patients with NB was previously reported to be shorter than that in the low TMB group (with three or fewer somatic variants) [112]. Further studies are required to clarify this relationship.

Thousands of somatic mutations are generated throughout the life cycle of cancer cells upon exposure to different sources of DNA damage, leaving a unique fingerprint specific to DNA damage. In the present study, we sought to identify a distinct mutational signature related to NB and the mutational processes underlying the observed signature (Figure 6). We observed a predominance of somatic C > T transition mutations in NB tumor samples, which are linked to multiple cancer types [113]. More importantly, the C > T mutational signature may be attributable to the DNA methylation of tumor suppressor genes [114]. In the current series, the clock-like COSMIC signatures SBS 5 and 1, which are ubiquitous in several human cancers, including NB, and correlate with patient age [115], were highly prevalent, followed by SBS 6 and 3. Because patients in our cohort were younger, these observed mutational signatures were not concordant, potentially indicating faster mutations in age-associated mutational patterns than usual and an imbalance between DNA damage and repair [116]. Similar to previous reports, the next most abundant signatures were SBS6 and SBS3, which were related to the failure of DNA mismatch repair and homologous recombination repair of double-stranded DNA, respectively. SBS3-associated mutations are predominantly observed in high-risk NB patients without *MYCN* amplification [117]. Our findings suggested that impaired DNA mismatch repair and homologous recombination of DNA damage repair underlie mutagenesis in NB. Unexpectedly, ROS-associated signature 18, which is highly enriched in NB, was not present in the NB tumor samples. In addition, a previous study showed a low prevalence of SBS18 in *MYCN* non-amplified NB [81]. These results correlate with a previous observation that this signature was frequently observed in patients with NB with known high risk and *MYCN* amplification [117]. Nonetheless, we did not observe a mutational signature 18 linked across the five NB patients with known high risk and *MYCN* amplification. The absence of signature 18 suggests differences in the approach or algorithm for acquiring mutational signatures, the genetic background of patients, the number of validated somatic mutations, the intrinsic and stable tendency of NB tumors to possess SBS18 [25], and more importantly, the non-specific manner of SBS18 to NB.

The most affected oncogenic signaling pathways in our NB tumor samples were RTK-RAS, NOTCH, and Hippo, which are linked to primary mutations in *ALK*, *NCOR2*, and *FAT2*, respectively. The RTK/RAS pathway has been implicated in cell differentiation, growth, progression, and survival, and its alterations are involved in carcinogenesis and high-risk NB [118]. A member of the RTK-RAS signaling pathway, for instance, the *ALK* gene, encodes a tyrosine kinase receptor belonging to the insulin receptor superfamily. As the most frequently mutated gene in neuroblastoma, *ALK* aberrations contribute to tumor growth, proliferation, and migration by activating tyrosine kinase receptors [119]. Numerous studies have shown that the NOTCH signaling pathway plays a role in neural proliferation, differentiation, and survival and possesses either oncogenic or tumor suppressor properties, depending on the type of cancer. This pathway has been shown to be involved in NB pathogenesis as a tumor suppressor that inhibits tumor cell differentiation [120]. NCOR2 is a nuclear receptor co-repressor 2 of the NOTCH signaling pathway that regulates the transcription of different tumors. NCOR2 has been shown to exert chromatin remodeling for the regulation of NB carcinogenesis [121]. The Hippo signaling pathway regulates cell proliferation, differentiation, apoptosis, and homeostasis in tissues and organs. A previous study demonstrated the significant role of the Hippo pathway in NB regulation and tumorigenesis, and the high expression of its members was associated with the invasion and metastasis of NB [122]. In accordance with other studies, somatic alterations in NB mainly affected the RTK-RAS signaling pathway [87].

GO and KEGG pathway enrichment analyses revealed potential functional classifications as well as pathways relevant to somatically mutated genes detected in our NB cohort. As a result, the GO enrichment analysis demonstrated that all mutated genes were subject to particular biological processes, including transcription regulation, chromatin remodeling, and the regulation of kinase activity. Consistently, genes related to MF were significantly enriched in tyrosine kinase activity and the transcriptional activity of factors, activators, and coactivators. Similar to our GO results, genes localized in recurrently deleted chromosomal regions are involved in the regulation of transcription [123], whereas genes in the low-risk group are linked to tyrosine kinase activity, as previously described [121]. Abnormalities in various transcriptional regulators and receptor tyrosine kinases contribute to the pathological development of neuroblastoma. For instance, the overexpression of the *MYCN* gene results in the inhibition of apoptotic signaling and induction of cell proliferation [124], and activating mutations in *ALK* promotes tumor growth, proliferation, and migration [119]. A previous study confirmed that loss of chromatin remodeling is related to NB progression [125]. Specifically, the enriched GO terms for all mutated genes were related to the regulation of gene expression. In addition to transcriptional regulation, the resulting KEGG analysis showed that the mutated genes were implicated in cancer and the PI3K-Akt signaling pathways. The latter promote cell survival in neuroblastoma and prevent apoptosis [126]. Hub genes are defined as those that interact with a large number of other genes. By constructing a PPI network and using CytoHubba, we identified *ATM*, *BRCA2*, *BRCA1*, *MLH1*, *ATRX*, *WRN*, *FANCM*, *RAD51C*, *CHEK2*, and *RAD51D* as the top 10 candidate hub genes associated with NB. ATM, a potential tumor suppressor in NB, plays a pivotal role in the regulation of apoptosis, cell cycle arrest, and repair of double-strand breaks caused by endogenous genomic stress or exogenous irradiation. A previous study demonstrated that the downregulation of *ATM* by miR-421 promotes neuroblastoma progression [127]. BRCA1 and BRCA2 are tumor suppressors in several cancers and have been implicated in DNA repair in response to DNA breaks, transcription regulation, and cell growth control. BRCA2 has been reported to be associated with NB development, although the underlying mechanisms are poorly understood. In *MYCN*-driven NB, BRCA1 is essential to maintain the transcription process and DNA repair of promoter sequences [128]. Further research is warranted to understand the exact roles of these hub genes in NB tumor development.

Driver mutations contribute to tumorigenesis primarily by affecting key cellular functions, including proliferation, apoptosis, cell growth, regulation of energy metabolism, and immune evasion [129]. The identification of new cancer driver mutations and genes may provide important insights into the genesis of NB and novel biomarkers for early diagnosis, prognosis, and targeted therapies for NB. In this study, we found that each NB tumor harbored approximately three driver mutations, which was very close to a previous report having approximately five driver mutations [130]. Among the 122 identified driver gene mutations, 5 were present in at least two samples, encompassing *MUC4*, *MUC16*, *ALK*, and *CTNND1*. These driver mutations included splice-site mutations in *MUC16*, an in-frame insertion of p.T1679_P1680ins, an in-frame deletion of p.L2662_D2709del in *MUC4*, an F1174L mutation in *ALK*, and an R439C mutation in *CTNND1*. The deleterious functional effects of recurrent driver mutations were predicted using several in silico prediction tools, and many of these tools classified these mutations as potentially having detrimental effects on protein function.

Although these alterations in the mucin proteins *MUC4* and *MUC16* have not been described previously in NB tumors, point mutations in these mucin genes have been identified in a previous study [81,87]. With the limitations of WES for the detection of indels (>20 bp) and splice sites, further confirmation of these mutations using hybrid WES and Sanger sequencing may be required to provide reliable results. The *ALK* F1174L mutation is frequently detected in familial or sporadic NB and is responsible for strong oncogenic activity in NB with amplified *MYCN*, induction of constitutive activation, and regulation of the *MYCN* gene [131]. In vitro studies on cell lines have shown that this mutation is sensitive to TAE684 and alectinib but confers resistance to crizotinib [132]. The tumor driver CTNND1 is the key component involved in cell–cell adhesion and Rac1, Cdc42, and Ras homolog gene family member A (RhoA) activation and has been reported to repress the migration and invasion of tumor cells [133]. Catenin potentially functions as either a tumor suppressor in human pancreatic cancer or an oncogene in hepatocellular carcinoma, whereby its overexpression promotes invasion and metastasis of HCC by modulating Wnt/β-catenin signaling [134]. Alternative splicing and altered localization of CTNND1 promotes cell migration and invasion [135]. In addition, *CTNND1* alterations are associated with poor OS [136]. Our findings revealed a novel recurrent driver mutation, R439C, in *CTNND1* of NB tumors, which may serve as a novel biomarker for early diagnosis and prognosis and as a therapeutic target.

From a clinical perspective, genomics-guided individualized precision therapy requires a robust biomarker to predict drug responsiveness and facilitate the successful treatment of patients. Herein, we identified 11 putative actionable biomarkers using the CGI, namely *NF1* Q1798*, Q2616*, and S636X, *ALK* F1174L and R1275Q, *SETD2* P10L and Q1829E, *BRCA1* R612S, *NOTCH1* D1670V, *ATR* S1372L, and *FGFR1* N577K, which were previously reported in NB or any cancer type and are likely to be drug targets of existing or currently being investigated in pre-clinical or clinical trials. Some of these mutations have been studied extensively for their roles in drug resistance. For example, the F1174L substitution appears to be one of the most common somatic mutations in *ALK* and is associated with *MYCN* amplification [137]. This alteration contributes to crizotinib resistance by promoting ATP binding, leading to decreased crizotinib binding [138]. For the *ALK* F1174L mutation, a high dose of crizotinib would be effective, in addition to alectinib [139] and a combination of crizotinib and the PI3K/AKT/mTOR pathway [140]. These previous findings are concordant with our results, showing that this mutation may confer susceptibility or resistance to crizotinib and susceptibility to alectinib. Because of its low ATP-binding affinity, R1275Q is one of the most frequent mutations in familial neuroblastoma and exhibits the highest sensitivity to crizotinib [141]. Moreover, it has been reported to be sensitive to TAE684 [142]. Only a handful of patients with NB contained candidate biomarkers for drug response due to a lack of validated variants of drug responses and the extensive intratumor heterogeneity in NB. As several drugs for the treatment of various cancer types are in the pre-clinical phase, they may have the potential to be repurposed as an alternative treatment for NB. This is further supported by the observation of potential responsiveness in patients with NB in our cohort to these drugs through their druggable alterations. Our findings demonstrate the use of a comprehensive database to predict the drug responsiveness of patients with putative cancer driver mutations, translating them into personalized medicine. In addition, it provides information on the frequency of druggable variants in patients with NB, which may be useful for drug development.

Despite intensive multimodal therapy, the long-term survival of high-risk patients remains poor. One of the main obstacles to the successful treatment of NB is chemoresistance. It has been reported that 71–85% of high-risk patients exhibit a partial or complete response after induction therapy, whereas approximately 20% show an unfavorable response to standard chemotherapy [13]. In our study, we investigated somatic variants associated with chemotherapy resistance in patients with NB. All five patients were resistant to chemotherapy. Correlation analysis identified variants in three genes, including *OR4N2*, *MC1R*, and NUP214, that were associated with chemotherapy resistance. The *SET*-*NUP214* (*TAF1*/*CAN*) fusion gene has shown a strong association with chemotherapy resistance in adult T-cell acute lymphoblastic leukemia [143]. However, it is important to note that the sample size is too small, which may result in low statistical power. Additional studies with larger sample sizes are necessary to confirm their association with chemoresistance.

To seek other therapeutic options for chemoresistant patients with NB, we employed the CGI platform to identify cancer driver mutations that are predictive of a favorable response to listed drugs. We found 14 driver mutations in 12 genes across all patients with chemoresistance (*n* = 5), all of which were associated with available drugs. *PALB2* (p.A245V) and *RAD51C* (p.S16G) mutations were present in two chemoresistant cases and were predictive of sensitivity to olaparib (a PARP inhibitor). This PARP inhibitor has been FDA-approved for the treatment of metastatic castration-resistant prostate cancer [144]. However, the repurposing of these FDA-approved drugs for NB treatment requires the assessment of their efficacy and safety in NB patients. Accordingly, further studies are necessary to evaluate the therapeutic potential of the drugs identified in our study for NB patients with chemoresistance.

Several limitations of the current study need to be addressed. First, the sample size was small, thereby limiting statistical power in the analyses, including the correlation analysis between clinicopathological parameters and mutations, and undermining the accuracy and reliability of the results. Because of the retrospective design, complete and exhaustive information about the patients could not be obtained from their medical records. Moreover, there were no matched normal samples for the subtraction of all germline variants when calling somatic mutations or for the further validation of somatic variants through gene expression analysis. A further limitation is the difficulty in excluding chemotherapy-induced mutations [145] and identifying somatic mutations associated with NB development and chemoresistance, as nearly all tumors were collected post-chemotherapy. However, we addressed these issues by identifying cancer driver mutations using the CGI server and comparing two groups of patients, those with chemosensitivity and those with chemoresistance, to find predictive markers for chemoresistance in NB. Another potential limitation is the highly heterogeneous nature of NB tumors [146]. Since only one area of the resected tumors was sampled and sent for whole-exome sequencing, this approach did not capture the complete picture of somatic mutations in those tumors, as sampling from different regions of the same tumor can yield different mutations. Analysis of the tumor immune microenvironment is not feasible due to the lack of complete and consistent histological data on both immune and non-immune cells across all tumor samples. Furthermore, functional studies of somatic driver mutations have not been performed to validate their role in the development of NB. Finally, most patients did not have their *MYCN* amplification status determined, as it was only assessed in cases where the pre-treatment risk classification by the pediatric oncologist was unclear. Despite these limitations, this study was able to show the somatic mutational landscape of patients with NB, identify predictive biomarkers for drug response, and provide therapeutic options for patients with chemotherapy resistance.

## 5. Conclusions

Our study is the first in Thai patients to draw a comprehensive mutational landscape of somatic mutations and identify frequently mutated genes, such as *MUC4* and *MUC16*, with significant cancer driver alterations. The recurrent missense mutation R439C in *CTNND1* has been identified as a novel putative cancer driver variant for NB. We demonstrated the utility of WES in predicting drug responses in patients with tumors, offering clinicians the right treatment. Promising drugs for the treatment of NB patients with chemotherapy resistance have been suggested. A significant correlation between gene mutation profiles and clinicopathological parameters of NB has been previously established. Mutations in *MUC4* and *LRRN3* showed a significant negative association with OS. The resulting TMB from tumor-only samples was inflated, and no differences were found between the high and low TMB groups in terms of the OS of patients. In the NB samples, the RTK-RAS, NOTCH, and Hippo signaling pathways were considerably affected, and all mutated genes were significantly enriched in the regulation of gene expression. Mutational signatures 5, 1, 6, and 3 were prominent, with the core genes relevant to NB being *ATM*, *BRCA2*, and *BRCA1*. However, some limitations were identified in our study, including small sample size, lack of matched normal samples, a good panel of normal samples made from the same library preparation kit and sequencing platform for calling somatic variants, and insufficient or lost follow-up of patients for survival analysis.

## Figures and Tables

**Table 1:** Clinicopathological features of patients with neuroblastoma (NB).

Characteristics	No. of Patient (*n* = 48) (%)
Sex	
Male	32 (66.7)
Female	16 (33.3)
Age at diagnosis (years)	
Mean ± SD	3.63 ± 3.41
Median (range)	2.7 (0.3–16.8)
<18 months	12 (25)
≥18 months	36 (75)
INRG stage	
L2	2 (4.2)
M	43 (89.6)
MS	3 (6.2)
INSS stage	
1	1 (2.1)
2A	1 (2.1)
3	1 (2.1)
4	43 (89.6)
4S	2 (4.1)
Pre-treatment risk group	
High risk	44 (91.6)
Standard risk	2 (4.2)
Low risk	2 (4.2)
MYCN amplification	
Yes	5 (10.4)
No	4 (8.3)
Unknown	39 (81.3)
Tumor size (cm), *n* = 43	
Mean ± SD	6.1 ± 3.2
Median (range)	5.0 (2.5–15.0)
Tumor histology	
Ganglioneuroblastoma	9 (18.8)
Ganglioneuroma	6 (12.5)
Neuroblastoma	30 (62.5)
Unknown	3 (6.2)
Primary site	
Adrenal	26 (54.2)
Extra-adrenal	22 (45.8)
Metastasis	
Yes	46 (95.8)
No	2 (4.2)
Metastatic site, *n* = 45	
BM	26 (57.8)
Bone	38 (84.4)
CNS	1 (2.2)
Liver	8 (17.8)
LN	6 (13.3)
Lung	7 (15.6)
Orbit	2 (4.4)
Skin	1 (2.2)
Spine	3 (6.7)
Recurrence	
Yes	5 (10.4)
No	43 (89.6)
Stage collection	
Pre-CM	2 (4.2)
Post-CM	43 (89.6)
Unknown	3 (6.2)
Response to treatment	
Complete response	27 (56.3)
Partial response	11 (22.9)
Stable disease	5 (10.4)
Unknown	5 (10.4)
Radiation	
Yes	6 (12.5)
No	39 (81.3)
Unknown	3 (6.2)
Vital status at last follow-up Alive	40 (83.3)
Dead	8 (16.7)
Vital status at 5 years	
Alive	17 (35.4)
Dead	31 (64.6)

INRG, International Neuroblastoma Risk Group; INSS, International Neuroblastoma Staging System; BM, bone marrow; CNS, central nervous system; LN, lymph node; CM, chemotherapy.

**Table 2:** List of known and novel recurrent somatic driver mutations predicted by Cancer Genome Interpreter.

Gene	Chr	Start	End	Ref	Alt	Amino Acid Change	COSMIC ID
*MUC4*	3	195,786,542	195,786,542	-	GGTGGCGTGACCTGTGGATACTGA GGAATTGTCGGTGACAGGAAGAG GGGTGGCGTGACCGGTGGATGCTG AGGAAGTGCTGGTGACAGGAAGA GA	p.T1679_P1680insSL PVTSTSSASTGHAT PLPVTDNSSVSTG HAT	NA
*MUC4*	3	195,783,453	195,783,596	GTCGGTGACAGGAAGAGAGGTGG TGTCACCTGTGGATGCTGAGGAAG TGTCGGTGACAGGAAGAGAGGTG GCATGACCGGTGGATGCTGAGGAA GGGCTAGTGACAGGAAGAGGCGT GGTGTCACCTGTGGATACTGAGGA AAG	-	p.L2662_D2709del	NA
*MUC16*	19	8,851,677	8,851,677	C	G	-	NA
*ALK*	2	29,220,829	29,220,829	G	T	p.F1174L	COSV66555460
*CTNND1*	11	57,802,091	57,802,091	C	T	p.R439C	COSV62385889

**Table 3:** List of recurrent cancer driver genes (known or predicted) detected in tumor samples of patients with NB.

Gene	Total Number of Mutations	Total Number of Samples (*n* = 48)	Frequency of Mutated Samples (%)
*ALK*	3	3	6.3
*BUB1B*	2	2	4.2
*CD209*	2	2	4.2
*CSMD3*	2	2	4.2
*CTNND1*	2	2	4.2
*ERBB4*	2	2	4.2
*ITGAV*	2	2	4.2
*KAT6A*	2	2	4.2
*KMT2C*	5	4	8.3
*MUC16*	2	2	4.2
*MUC4*	14	12	25.0
*MYH11*	2	2	4.2
*NF1*	3	2	4.2
*POLQ*	2	2	4.2
*PREX2*	4	4	8.3
*SETD2*	2	2	4.2

**Table 4:** Predictive biomarkers of drug response in NB.

Gene	Alteration	Drug	Diseases	Response	Evidence
*NF1*	Q1798* and Q2616*	Retinoic acids	Neuroblastoma	Resistant	D
*NF1*	Q1798* and Q2616*	Cobimetinib + trametinib	Any cancer type	Responsive	D
*NF1*	S636X	Retinoic Acids	Neuroblastoma	Resistant	D
*NF1*	S636X	Cobimetinib + Trametinib	Any cancer type	Responsive	D
*ALK*	F1174L	Crizotinib	Neuroblastoma	Resistant	D
*ALK*	F1174L	Crizotinib	Neuroblastoma	Responsive	D
*ALK*	F1174L	Crizotinib	Neuroblastoma	Responsive	C
*ALK*	F1174L	Alectinib	Neuroblastoma	Responsive	D
*ALK*	F1174L	TAE684	Neuroblastoma	Responsive	D
*ALK*	F1174L	AZD3463	Neuroblastoma	Responsive	D
*ALK*	F1174L	Lorlatinib	Neuroblastoma	Responsive	D
*ALK*	R1275Q	TAE684	Neuroblastoma	Resistant	D
*ALK*	R1275Q	TAE684	Neuroblastoma	Responsive	D
*ALK*	R1275Q	Crizotinib	Neuroblastoma	Responsive	D
*ALK*	R1275Q	Crizotinib	Neuroblastoma	Responsive	C
*ALK*	R1275Q	Lorlatinib	Neuroblastoma	Responsive	D
*SETD2*	P10L	WEE1 inhibitors	Any cancer type	Responsive	D
*SETD2*	Q1829E	WEE1 inhibitors	Any cancer type	Responsive	D
*BRCA1*	R612S	WEE1 inhibitors	Any cancer type	Responsive	C
*NOTCH1*	D1670V	Gamma secretase inhibitors (Ro4929097,Pf-03084014,Mk-0752,etc)	Any cancer type	Responsive	C
*ATR*	S1372L	Olaparib (PARP inhibitor)	Ovary, Any cancer type	Responsive	D
*FGFR1*	N577K	Azd4547 + Nvp-Bgj398 + Erdafitinib + 1265229-25-1	Any cancer type	Responsive	D

Note: “C” denotes early trials and “D” denotes pre-clinical. Asterisk denotes a truncating mutation.

## Data Availability

The data presented in this study are available in the main text or the Supplementary Materials, with additional data accessible upon request from the corresponding author due to ethical concerns regarding patient privacy.

## Supplementary Materials

The following supporting information can be downloaded at https://www.mdpi.com/article/10.3390/jpm14090950/s1. Supplementary Figure S1: Distribution of variant allele frequencies (VAF) in the top 10 frequently mutated genes. Supplementary Figure S2: Distribution of the six classes of single base substitutions (SBS) generated by Mutalisk. Supplementary Figure S3: Sanger sequencing validation of somatic mutations identified in *CTNND1*. The top panel shows sequencing traces from a blood sample of a non-neuroblastoma patient, serving as a control (sample 67042), while the two lower panels display sequencing traces from tumor samples analyzed in this study (samples A040 and A140). All samples were obtained from different patients. Supplementary Table S1: List of our customized cancer-associated genes. Supplementary Table S2: Details of somatic single-nucleotide variants and indels in the NB cohort. Supplementary Table S3: Correlation of clinicopathological features with the mutational status of the top 10 frequently mutated genes. Supplementary Table S4: List of driver and passenger mutations identified using Cancer Genome Interpreter (CGI). Supplementary Table S5: Details of predictive biomarkers of drug response identified by the CGI. Supplementary Material: Membership of the Thai Pediatric Cancer Atlas (TPCA) Consortium.
