# Supplementary material for: Whole-Exome Sequencing Reveals Novel Candidate Driver Mutations and Potential Druggable Mutations in Patients with High-Risk Neuroblastoma
Source: J Pers Med. 2024 Sep 8;14(9):950. doi: 10.3390/jpm14090950 (PMC11433071; doi:10.3390/jpm14090950)
Supplement: Supplementary file 1 [file jpm-14-00950-s001.zip › Supplementary Figure S1.pdf]

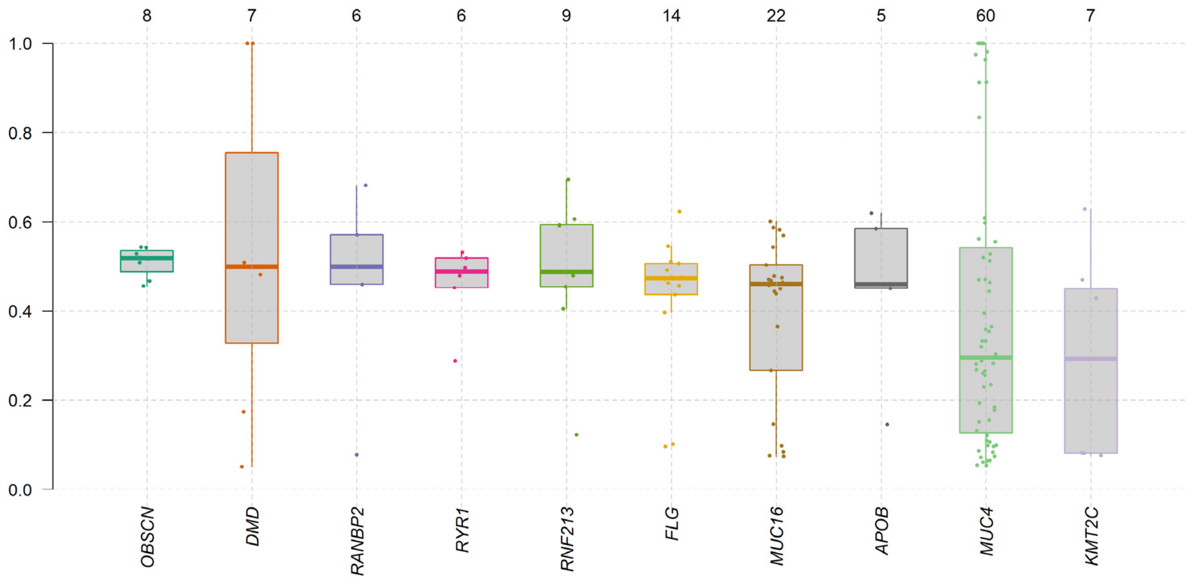

Supplementary Figure S1. Distribution of variant allele frequencies (VAF) in the top 10 frequently mutated genes. Genes are ordered by median VAF. The numbers on the top indicate the number of mutations.
