# Supplementary material for: Whole-Exome Sequencing Reveals Novel Candidate Driver Mutations and Potential Druggable Mutations in Patients with High-Risk Neuroblastoma
Source: J Pers Med. 2024 Sep 8;14(9):950. doi: 10.3390/jpm14090950 (PMC11433071; doi:10.3390/jpm14090950)
Supplement: Supplementary file 1 [file jpm-14-00950-s001.zip › Supplementary Figure S2.pdf]

row min — row max

id	C>A	C>G	C>T	T>A	T>C	T>G
A015	11.3	10.6	42.7	5.1	23.4	7.0
A035	12.9	10.2	44.0	4.2	22.2	6.5
A040	13.9	11.4	43.5	4.7	21.7	4.7
A045	11.6	11.1	44.3	4.3	22.8	5.9
A049	12.9	11.8	46.1	4.2	19.9	5.0
A058	12.1	10.2	46.8	5.1	19.8	6.2
A074	11.0	10.1	45.2	6.0	21.8	6.0
A075	13.6	11.6	42.6	6.1	20.9	5.3
A096	12.0	10.3	44.8	4.3	23.5	5.1
A112	12.6	11.7	42.7	5.4	21.7	5.9
A131	10.9	11.7	44.1	5.5	22.6	5.1
A140	9.5	12.3	45.7	5.2	21.4	6.0
A166	13.0	12.6	43.5	4.5	19.6	6.7
A174	12.8	11.1	42.6	5.5	21.6	6.5
A197	11.8	11.2	49.1	3.8	19.4	4.6
A234	11.1	10.9	42.7	5.3	22.6	7.2
A241	13.2	9.5	47.2	4.4	20.0	5.7
A248	10.9	9.6	45.9	4.8	21.4	7.3
A254	10.7	10.7	42.5	6.1	22.6	7.4
A279	12.2	11.5	44.9	4.4	20.6	6.4
A292	12.2	11.0	48.4	5.3	18.4	4.8
A297	12.1	11.1	44.0	3.4	22.9	6.5
A303	12.3	9.6	48.8	4.2	19.4	5.7
A309	9.8	10.8	47.7	3.4	21.7	6.5
A330	11.0	12.0	44.4	4.0	21.8	6.9
A374	13.0	11.1	45.2	5.6	19.0	6.0
A398	10.5	10.8	44.2	6.1	23.1	5.3
A400	11.9	11.7	45.3	4.5	21.7	5.0
A402	10.7	11.1	44.6	5.5	22.2	5.8
A404	12.1	11.1	43.0	5.6	22.8	5.4
A410	11.7	11.7	44.4	5.1	21.7	5.4
A412	8.9	11.8	46.3	4.9	23.0	5.2
A420	14.2	11.8	43.9	5.5	19.8	4.9
A424	11.1	11.7	47.2	5.1	20.1	4.8
A434	13.8	11.2	44.6	4.7	20.2	5.5
A439	9.2	11.6	44.6	4.5	25.4	4.7
A442	11.9	11.3	45.2	5.0	20.8	5.8
A461	13.5	9.7	43.3	5.5	22.4	5.5
A462	13.7	11.8	43.1	4.3	20.6	6.5
A474	11.7	10.7	44.6	5.2	20.1	7.7
A492	11.5	11.8	45.9	4.2	21.5	5.1
A497	9.8	10.9	44.2	4.9	23.6	6.6
A518	12.6	9.6	43.2	4.5	23.1	6.9
A520	11.4	9.7	46.7	4.3	21.1	6.7
A543	11.4	11.6	43.9	6.2	21.6	5.2
DM004	14.7	11.1	40.9	5.7	20.5	7.1
DM014	12.7	10.7	39.3	4.2	26.8	6.3
DM016	12.6	11.6	44.7	5.4	19.3	6.4

Supplementary Figure S2. Distribution of the six classes of single base substitutions (SBS) generated by Mutalisk. Columns represent the six types of SBS, and rows indicate the samples. The numbers in matrix represent the percentage of each type of SBS, with darker brown indicating a higher percentage of that specific SBS.
