# Supplementary material for: Whole-Exome Sequencing Reveals Novel Candidate Driver Mutations and Potential Druggable Mutations in Patients with High-Risk Neuroblastoma
Source: J Pers Med. 2024 Sep 8;14(9):950. doi: 10.3390/jpm14090950 (PMC11433071; doi:10.3390/jpm14090950)
Supplement: Supplementary file 1 [file jpm-14-00950-s001.zip › Supplementary Figure S3.pdf]

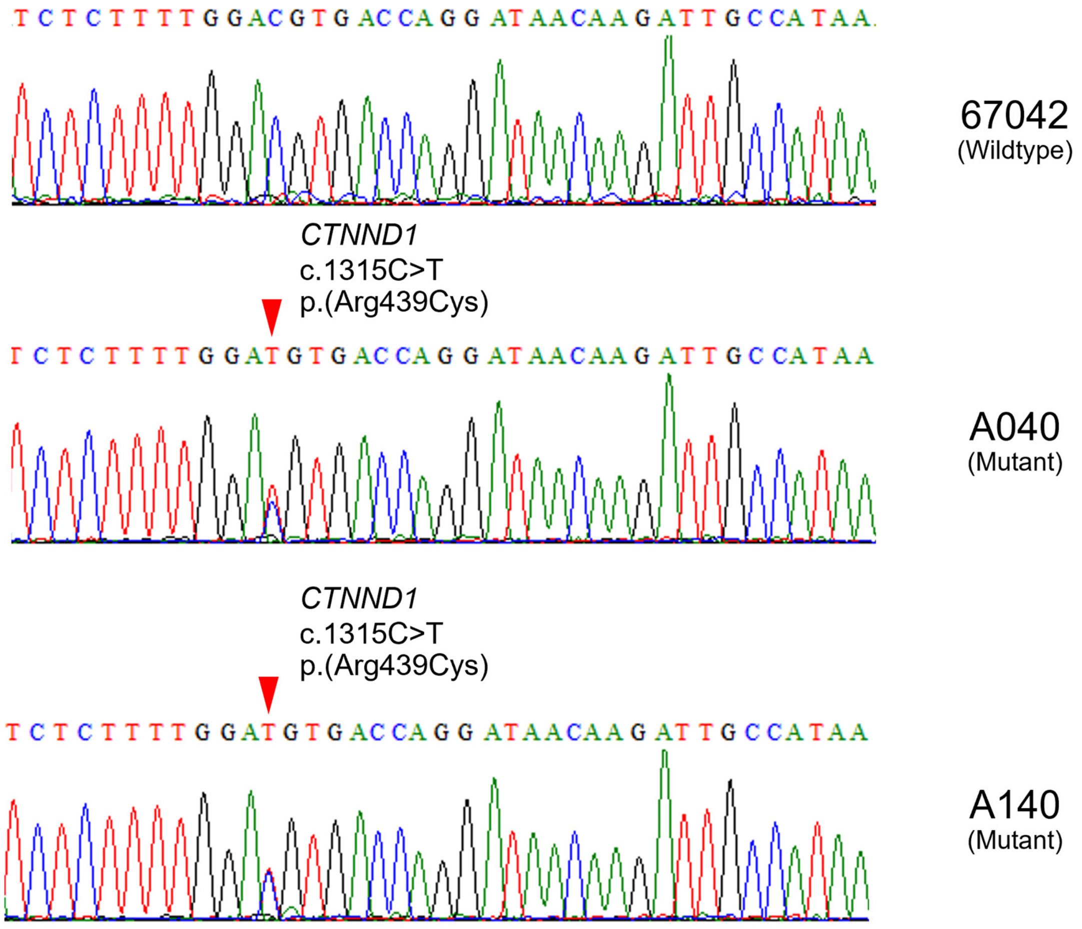

Supplementary Figure S3. Sanger sequencing validation of somatic mutations identified in *CTNND1*. The top panel shows sequencing traces from a blood sample of a non-neuroblastoma patient, serving as a control (sample 67042), while the two lower panels display sequencing traces from tumor samples analyzed in this study (samples A040 and A140). All samples were obtained from different patients.
