# Supplementary material for: Whole-Exome Sequencing Reveals Novel Candidate Driver Mutations and Potential Druggable Mutations in Patients with High-Risk Neuroblastoma
Source: J Pers Med. 2024 Sep 8;14(9):950. doi: 10.3390/jpm14090950 (PMC11433071; doi:10.3390/jpm14090950)
Supplement: Supplementary file 1 [file jpm-14-00950-s001.zip › Supplementary Material.pdf]

Thai Pediatric Cancer Atlas (TPCA) Consortium Author List:

Faculty of Medicine Ramathibodi Hospital:

1) **Suradej Hongeng** – Division of Hematology and Oncology, Department of Pediatrics, Faculty of Medicine Ramathibodi Hospital, Mahidol University, Bangkok 10400, Thailand.

2) **Usanarat Anurathapan** – Division of Hematology and Oncology, Department of Pediatrics, Faculty of Medicine Ramathibodi Hospital, Mahidol University, Bangkok 10400, Thailand.

3) **Wararat Chiangjong** – Pediatric Translational Research Unit, Department of Pediatrics, Faculty of Medicine Ramathibodi Hospital, Mahidol University, Bangkok 10400, Thailand.

4) **Praewa Suthapot** – Division of Hematology and Oncology, Department of Pediatrics, Faculty of Medicine Ramathibodi Hospital, Mahidol University, Bangkok 10400, Thailand.

5) **Somchai Chutipongtanate** – Division of Epidemiology, Department of Environmental and Public Health Sciences, University of Cincinnati College of Medicine, Cincinnati, OH 45267, USA.

6) **Nutkridta Pongsakul** – Pediatric Translational Research Unit, Department of Pediatrics, Faculty of Medicine Ramathibodi Hospital, Mahidol University, Bangkok 10400, Thailand.

7) **Jirawan Weeraphan** – Division of Hematology and Oncology, Department of Pediatrics, Faculty of Medicine Ramathibodi Hospital, Mahidol University, Bangkok 10400, Thailand.

8) **Nilubon Singhto** – Ramathibodi Comprehensive Cancer Center, Faculty of Medicine Ramathibodi Hospital, Mahidol University, Bangkok 10400, Thailand.

9) **Natini Jinawath** – Ramathibodi Comprehensive Cancer Center, Faculty of Medicine Ramathibodi Hospital, Mahidol University, Bangkok 10400, Thailand.

Faculty of Medicine, Prince of Songkla University:

1) **Surasak Sangkhathat** – Department of Biomedical Sciences and Biomedical Engineering, Faculty of Medicine, Prince of Songkla University, Songkhla 90110, Thailand.

2) **Pongsakorn Choochuen** – Department of Biomedical Sciences and Biomedical Engineering, Faculty of Medicine, Prince of Songkla University, Songkhla 90110, Thailand.

3) **Natakorn Nokchan** – Department of Biomedical Sciences and Biomedical Engineering, Faculty of Medicine, Prince of Songkla University, Songkhla 90110, Thailand.

4) **Natthapon Khongcharoen** – Department of Biomedical Sciences and Biomedical Engineering, Faculty of Medicine, Prince of Songkla University, Songkhla 90110, Thailand.

5) **Wanwisa Maneechay** – Innovation Center, Faculty of Medicine, Prince of Songkla University, Songkhla 90110, Thailand.

6) **Yanisa Klaewtanong** – Translational Medicine Research Center, Faculty of Medicine, Prince of Songkla University, Songkhla 90110, Thailand.

7) **Wararak Kaewrattana** – Department of Biomedical Sciences and Biomedical Engineering, Faculty of Medicine, Prince of Songkla University, Songkhla 90110, Thailand.

Faculty of Medicine, Chiang Mai University:

1) **Dumnoensun Pruksakorn** – Center of Multidisciplinary Technology for Advanced Medicine (CMUTEAM), Faculty of Medicine, Chiang Mai University, Chiang Mai 50200, Thailand.

2) **Parunya Chaiyawat** – Center of Multidisciplinary Technology for Advanced Medicine (CMUTEAM), Faculty of Medicine, Chiang Mai University, Chiang Mai 50200, Thailand.

3) **Jeerawan Klangjorhor**

– Center of Multidisciplinary Technology for Advanced Medicine (CMUTEAM), Faculty of Medicine, Chiang Mai University, Chiang Mai 50200, Thailand.

– Office of research administration, Chiang Mai University, Chiang Mai 50200, Thailand.

4) **Nutnicha Sirikaew** – Department of Biochemistry, Faculty of Medicine, Chiang Mai University, Chiang Mai 50200, Thailand.

5) **Petlada Yongpitakwattana** – Center of Multidisciplinary Technology for Advanced Medicine (CMUTEAM), Faculty of Medicine, Chiang Mai University, Chiang Mai 50200, Thailand.

6) **Sutpirat Moonmuang**

– Center of Multidisciplinary Technology for Advanced Medicine (CMUTEAM), Faculty of Medicine, Chiang Mai University, Chiang Mai 50200, Thailand.

– Office of research administration, Chiang Mai University, Chiang Mai 50200, Thailand.

7) **Tanyaluck Kampoun** – Center of Multidisciplinary Technology for Advanced Medicine (CMUTEAM), Faculty of Medicine, Chiang Mai University, Chiang Mai 50200, Thailand.

8) **Viraporn Thepbundit** – Department of Biochemistry, Faculty of Medicine, Chiang Mai University, Chiang Mai 50200, Thailand

9) **Sasimol Udomruk** – Center of Multidisciplinary Technology for Advanced Medicine (CMUTEAM), Faculty of Medicine, Chiang Mai University, Chiang Mai 50200, Thailand.

10) **Santhasiri Orrapin** – Center of Multidisciplinary Technology for Advanced Medicine (CMUTEAM), Faculty of Medicine, Chiang Mai University, Chiang Mai 50200, Thailand

11) **Warunyoo Phannasorn** – Center of Multidisciplinary Technology for Advanced Medicine (CMUTEAM), Faculty of Medicine, Chiang Mai University, Chiang Mai 50200, Thailand

12) **Thanapak Jaimalai** – Center of Multidisciplinary Technology for Advanced Medicine (CMUTEAM), Faculty of Medicine, Chiang Mai University, Chiang Mai 50200, Thailand

13) **Pimpisa Teeyakasem** – Research Administration Section, Faculty of Medicine, Chiang Mai University, Chiang Mai 50200, Thailand

Co- chairs: Suradej Hongeng[1], Usanarat Anurathapan[1], Somchai Chutipongtanate[3], Surasak Sangkhathat[5,7,8], Dumnoensun Pruksakorn[9]

Tissue and clinical data source sites: **Mahidol University** Usanarat Anurathapan[1], Natini Jinawath[4], Praewa Suthapot[1,5,9], Nilubon Singhto[4]; **Prince of Songkla University** Surasak Sangkhathat[5,7,8], Pongsakorn Choochuen[5], Wanwisa Maneechay[6], Yanisa Klaewtanong[7], Wararak Kaewrattana[5]; **Chiang Mai University** Dumnoensun Pruksakorn[9], Pimpisa Teeyakasem[12]

Cancer multi- omics data centers: **Mahidol University** Wararat Chiangjong[2], Praewa Suthapot[1,5,9], Jirawan Weeraphan[1], Nutkridta Pongsakul[2]; **Prince of Songkla University** Pongsakorn Choochuen[5], Natakorn Nokchan[5], Natthapon Khongcharoen[5]; **Chiang Mai University** Parunya Chaiyawat[9], Nutnicha Sirikaew[11], Viraporn Thepbundit[11], Petlada Yongpitakwattana[9]

Data analysis subgroup: **Mahidol University** Wararat Chiangjong[2], Praewa Suthapot[1,5,9], Jirawan Weeraphan[1], Nutkridta Pongsakul[2]; **Prince of Songkla University** Pongsakorn Choochuen[5], Natakorn Nokchan[5], Natthapon Khongcharoen[5]; **Chiang Mai University** Nutnicha Sirikaew[11], Jeerawan Klangjorhor[9,10], Sutpirat Moonmuang[9,10], Tanyaluck Kampoun[9], Viraporn Thepbundit[11], Sasimol Udomruk[9], Santhasiri Orrapin[9], Warunyoo Phannasorn[9], Thanapak Jaimalai[9]

[1] Division of Hematology and Oncology, Department of Pediatrics, Faculty of Medicine Ramathibodi Hospital, Mahidol University. [2] Pediatric Translational Research Unit, Department of Pediatrics, Faculty of Medicine Ramathibodi Hospital, Mahidol University. [3] Division of Epidemiology, Department of Environmental and Public Health Sciences, University of Cincinnati College of Medicine. [4] Ramathibodi Comprehensive Cancer Center, Faculty of Medicine Ramathibodi Hospital, Mahidol University. [5] Department of Biomedical Sciences and Biomedical Engineering, Faculty of Medicine, Prince of Songkla University. [6] Innovation Center, Faculty of Medicine, Prince of Songkla University. [7] Translational Medicine Research Center, Faculty of Medicine, Prince of Songkla University. [8] Department of Surgery, Faculty of Medicine, Prince of Songkla University. [9] Center of Multidisciplinary Technology for Advanced Medicine (CMUTEAM), Faculty of Medicine, Chiang Mai University. [10] Office of research administration, Chiang Mai University. [11] Department of Biochemistry, Faculty of Medicine, Chiang Mai University. [12] Research Administration Section, Faculty of Medicine, Chiang Mai University.
